# Transcript and metabolite analysis in Trincadeira cultivar reveals novel information regarding the dynamics of grape ripening

**DOI:** 10.1186/1471-2229-11-149

**Published:** 2011-11-02

**Authors:** Ana M Fortes, Patricia Agudelo-Romero, Marta S Silva, Kashif Ali, Lisete Sousa, Federica Maltese, Young H Choi, Jerome Grimplet, José M Martinez- Zapater, Robert Verpoorte, Maria S Pais

**Affiliations:** 1 Plant Systems Biology Lab, Departmento de Biologia Vegetal/ICAT, Center for Biodiversity, Functional and Integrative Genomics (BioFIG), FCUL, 1749-016 Lisboa, Portugal; 2 Centro de Química e Bioquímica, Departamento de Química e Bioquímica, FCUL, Lisbon, Portugal; 3 Natural Products Laboratory, Institute of Biology, Leiden University, 2300 RA Leiden, The Netherlands; 4 Department of Statistics and Operational Research, CEAUL (Centro de Estatística e Aplicações da UL), FCUL, Lisbon, Portugal; 5 Instituto de Ciencias de la Vid y del Vino (CSIC, UR, Gobierno de La Rioja), CCT, C/Madre de Dios 51, 26006 Logroño, Spain

## Abstract

**Background:**

Grapes (*Vitis vinifera *L.) are economically the most important fruit crop worldwide. However, the complexity of molecular and biochemical events that lead to the onset of ripening of nonclimacteric fruits is not fully understood which is further complicated in grapes due to seasonal and cultivar specific variation. The Portuguese wine variety Trincadeira gives rise to high quality wines but presents extremely irregular berry ripening among seasons probably due to high susceptibility to abiotic and biotic stresses.

**Results:**

Ripening of Trincadeira grapes was studied taking into account the transcriptional and metabolic profilings complemented with biochemical data. The mRNA expression profiles of four time points spanning developmental stages from pea size green berries, through *véraison *and mature berries (EL 32, EL 34, EL 35 and EL 36) and in two seasons (2007 and 2008) were compared using the Affymetrix GrapeGen^® ^genome array containing 23096 probesets corresponding to 18726 unique sequences. Over 50% of these probesets were significantly differentially expressed (1.5 fold) between at least two developmental stages. A common set of modulated transcripts corresponding to 5877 unigenes indicates the activation of common pathways between years despite the irregular development of Trincadeira grapes. These unigenes were assigned to the functional categories of "metabolism", "development", "cellular process", "diverse/miscellanenous functions", "regulation overview", "response to stimulus, stress", "signaling", "transport overview", "xenoprotein, transposable element" and "unknown". Quantitative RT-PCR validated microarrays results being carried out for eight selected genes and five developmental stages (EL 32, EL 34, EL 35, EL 36 and EL 38). Metabolic profiling using ^1^H NMR spectroscopy associated to two-dimensional techniques showed the importance of metabolites related to oxidative stress response, amino acid and sugar metabolism as well as secondary metabolism. These results were integrated with transcriptional profiling obtained using genome array to provide new information regarding the network of events leading to grape ripening.

**Conclusions:**

Altogether the data obtained provides the most extensive survey obtained so far for gene expression and metabolites accumulated during grape ripening. Moreover, it highlighted information obtained in a poorly known variety exhibiting particular characteristics that may be cultivar specific or dependent upon climatic conditions. Several genes were identified that had not been previously reported in the context of grape ripening namely genes involved in carbohydrate and amino acid metabolisms as well as in growth regulators; metabolism, epigenetic factors and signaling pathways. Some of these genes were annotated as receptors, transcription factors, and kinases and constitute good candidates for functional analysis in order to establish a model for ripening control of a non-climacteric fruit.

## Background

Grapes (*Vitis *species) are economically the most important fruit crop worldwide with a global production of around 67 million tons in 2008 (FAOSTAT, 2011). Moreover, the consumption of table grapes and wine has numerous nutritional and health benefits for humans due to antioxidant polyphenols such as resveratrol [1]. Grape seeds have significant content of phenolic compounds such as gallic acid, catechin and epicatechin, and a wide variety of proanthocyanidins which show significant cancer prevention potential [2]. Red wines contain more than 200 polyphenolic compounds that are thought to act as antioxidants. In particular, resveratrol exhibits cardioprotective effects and anticancer properties [2].

In traditional wine areas, the production should present typicity that is dependent on grapevine variety among other factors. Therefore, wine improvement is greatly limited to the natural variability of the cultivars. In this respect, less known Portuguese and Spanish cultivars offer plenty of choice to develop wines with different characteristics that may constitute a competitive advantage in a demanding global market. Among these varieties is the Portuguese Trincadeira which presents irregular ripening in different seasons and is extremely sensitive to *Botrytis *sp, and *Plasmopara viticola *but often gives rise to unique wines (Jorge Böhm, Plansel, personal communication).

In contrast to the well studied climacteric fruits such as tomato, the process of development and ripening of non-climacteric fruits such as grapes is less investigated. Grape berry development consists of two successive sigmoidal growth periods separated by a lag phase; from anthesis to ripening it can be divided into three major phases [3] with more detailed descriptive designations, known as the modified E-L system, being used to define more precise growth stages over the entire grapevine lifecycle [4]. The first growth period corresponds to the formation of the seed embryos and the pericarp. The first stage is characterized by exponential growth of the berry, biosynthesis of tannins and hydroxycinnamic acids, and accumulation of two organic acids, tartrate and malate. Tannins are present in skin and seed tissues and nearly absent in the flesh, and are responsible for the bitter and astringent properties of red wine. The onset of ripening, *véraison*, constitutes a transition phase during which growth declines and there is initiation of colour development (anthocyanin accumulation in red grapes) and berry softening. Ripening (the last phase) is characterized by an increase in pH, additional berry growth mainly due to cell expansion and accumulation of soluble sugars, cations such as potassium and calcium, anthocyanins and flavour-enhancing compounds.

The many chemical compounds contributing to flavour (taste and aroma) in wines are determined in the vineyard by factors such as the natural environment, vineyard management practices, and vine genotypes, among others. A better understanding of accumulation of sugars and flavour compounds in the berry is of critical importance to adjust grape growing practices to market needs. Increased knowledge of grape ripening will help on establishing optimal grape maturity for harvest which is difficult to determine due to the tremendous variability in ripening between berries within a grape cluster. Moreover, it will contribute to maintain a sustainable production of high quality grapes in a changing environment, one major challenge for viticulture in this century.

Molecular evidence is lacking for a single master switch controlling ripening initiation, such as the established role for ethylene in climacteric fruit ripening. It is known that following *véraison *stage, auxin and cytokinin contents decrease while abscisic acid concentration increases [5,6]. Abscisic acid, brassinosteroids, and, to a lesser extent, ethylene, have been implicated in control of fruit ripening initiation in grapevine but their modes of action at the molecular level require further clarification [7-10]. Moreover, certain growth regulators such as polyamines have been little studied in the context of grape ripening.

The availability of high-throughput analysis methods and a high quality draft of the grapevine genome sequence [11,12], together with studies on transcriptomics [13-16], proteomics [17-19] and metabolic profiling [20] contributed to greatly increase the knowledge on grape ripening. Moreover, genetic maps have been developed enabling the identification of QTLs for important traits and a consensus map has been built [21].

This work describes the first comprehensive transcriptional and metabolic analysis of grape ripening performed over two seasons (2007 and 2008). Transcriptional profiling was carried out using the second generation of Affymetrix Vitis microarrays (GRAPEGEN GenChip) that covers approximately 50% of the genome, and taking into account both genomic annotation based on 12X coverage grapevine genome sequence assembly and EST homology- based annotation. Information regarding the current model of grapes' ripening is confirmed and new information is provided that may be cultivar specific since little is known about this process in other Vitis grapevine cultivars.

## Results and Discussion

### Phenotypic and metabolic characterization of berries

**Figure 1:**
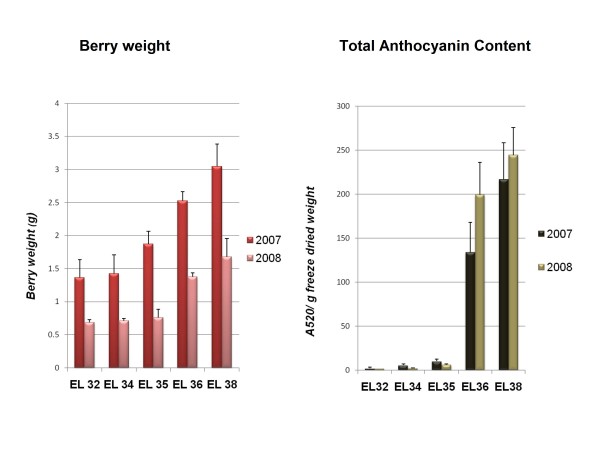
**Fresh berry weight (g) and total anthocyanin content expressed as absorbance at 520 nm per g of freeze dried material**. Bars represent standard variation.

**Figure 2:**
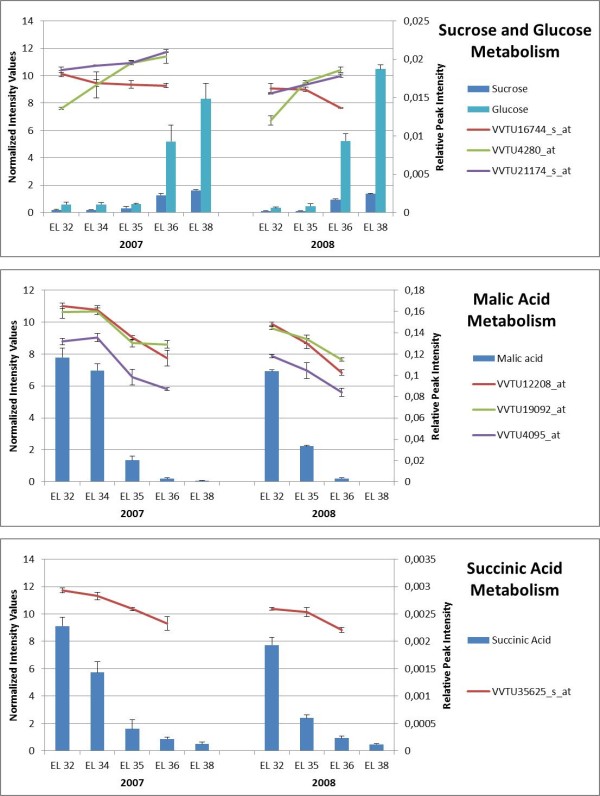
**Metabolism of sucrose, glucose, malic acid and succinic acid: gene expression and metabolite content**. Relative quantification of sucrose, α-glucose, malic acid and succinic acid is based on characteristic chemical shift (*δ *5.39, *δ *5, 17, *δ *2.67 and *δ *2, 62, respectively), and corresponding peak intensity. Malate and succinate contents are higher at pre-*véraison *stages peaking at EL 32 whereas contents in sucrose and α-glucose increase at post-*véraison *stages reaching maximal levels at EL 38. Expression levels of genes coding for sucrose synthase (VVTU16744_s_at), sucrose-phosphate synthase 1 (VVTU4280_at), sucrose phosphatase (VVTU21174_s_at), phosphoenolpyruvate carboxylases (VVTU12208_at, VVTU19092_at), glyoxysomal precursor of malate dehydrogenase (VVTU4095_at), succinate-semialdehyde dehydrogenase (VVTU35625_s_at) are based on microarray.

Grape berries were sampled at five developmental stages according to E-L system [4] during 2007 and 2008 growing seasons, and taking into account berry weight, organic acids, sugars and anthocyanin content (Figures 1, 2). These developmental stages were identified as EL 32 characterized by small hard green berries accumulating organic acids; EL 34 just before *véraison *characterized by green berries, which are starting to soften (this stage was considered for all analyses only in 2007); EL 35 corresponding to *véraison*; EL 36 involving sugar and anthocyanins accumulation, and active growth due to cell enlargement; and EL 38 corresponding to harvest time. The date of *véraison *was set at approximately 9 weeks post-anthesis in both years. However, berry development was very irregular (e.g. berry size) when the two years are compared probably due to different precipitation patterns (Additional File 1) and genotypic characteristics of Trincadeira. Irregular grape ripening has been observed for this cultivar in previous years (unpublished). Berry weight was not increased from EL 32 until EL 36 in 2008. Furthermore, the considerable difference in anthocyanin content between the two consecutive years at EL 36 may be mostly due to the fact that berries growing during the 2008 season did not expand as in 2007. In fact, berry weight almost doubled in the later season (Figure 1). Thus, the percentage of skin per berry was higher in 2008, which might account for an increase in anthocyanin content. In addition, environmental factors such as water stress may also be involved [22].

Additional metabolic profiling of Trincadeira grapes was carried out using ^1^H NMR. Signals at *δ *5.39 (d, *J *= 3.9 Hz), *δ *5, 17 (d, J = 3.5 Hz), *δ *2.67 (dd, J = 16.0, 7.0 Hz) and *δ *2.62 (s) were assigned to be anomeric proton of glucose moiety of sucrose, anomeric proton of α- and β-glucose, malic acid and succinic acid, respectively (Table 1). These chemical shifts were selected for relative quantification (based on signal integration normalized to internal standard) of these metabolites during ripening as shown in Figure 2.

**Table 1:** List of metabolites identified by ^1^H NMR and two dimensional NMR experiments.

Metabolite	Chemical shift	Multiplicity/Coupling constant
***cis*- Caffeoyl derivative**	δ 5.91	(d, J = 13.0 Hz)

	δ 6.89	(d, J = 8.5 Hz)

	δ 6.95	(d, J = 13.0 Hz)

	δ 7.56	(d, J = 8.5 Hz)

***cis*-Coumaroyl derivative**	δ 5.93	(d, J = 13.0 Hz)

	δ 6.83	(d, J = 9.5 Hz)

	δ 7.02	(d, J = 13.0 Hz)

	δ 7.58	(d, J = 9.5 Hz)

***trans*-caftaric acid (caffeic acid conjugated with tartaric acid)**	δ 7.64/δ 7.15	(d, J = 16.0 Hz)/(d, J = 2.0 Hz)

	δ 7.07	(dd, J = 8.5 Hz, 2.0 Hz)

	δ 6.88	(d, J = 8.5 Hz)

	δ 6.38	(d, J = 16.0 Hz)

	δ 5.51	(s)

**Sucrose**	δ 5.39	(d, J = 3.9 Hz)

**α-Glucose**	δ 5.17	(d, J = 3.5 Hz)

**β-Glucose**	δ 4.56	(d, J = 7.5 Hz)

**Tartaric acid**	δ 4.50	(s)

**Malic acid**	δ 2.67	(dd, J = 16.0, 7.0)

	δ 2.82	(dd, J = 16.0, 4.5)

	δ 4.43	(dd, J = 7.0, 4.5)

**Choline**	δ 3.22	(s)

**Citric acid**	δ 2.93	(d, J = 16.0 Hz)

	δ 2.76	(d, J = 16.0 Hz)

**Succinic acid**	δ 2.62	(s)

**Proline**	δ 2.35	(m)

	δ 3.37	(m)

**Glutamate**	δ 2.44	(td, J = 16.2, 7.5)

	δ 2.13	(m)

**Acetic acid**	δ 1.91	(s)

**Arginine**	δ 1.92	(m)

	δ 1.72	(m)

**Alanine**	δ 1.48	(d, J = 7.4 Hz)

**Threonine**	δ 1.32	(d, J = 6.5 Hz)

**ethyl-β -glucoside**	δ 1.21	(t, J = 7)

**Valine**	δ 1.06	(d, J = 7.0 Hz)

	δ 1.01	(d, J = 7.0 Hz)

**Leucine**	δ 0.96	(d, J = 7.5)

	δ 0.98	(d, J = 7.5)

	Trace amounts	

**γ-Aminobutyric acid (GABA)**	δ 1.90	(m)

	δ 2.31	(t, J = 7.5)

	δ 3.01	(t, J = 7.5)

**α-Linolenic acid**	δ 0.95	(t, J = 7.5)

	Trace amounts	

**Gallic acid**	δ 7.03	(s)

	Trace amounts	

**Ascorbic acid**	δ 4.59	(d. J = 2.0 Hz)

**Syringic acid**	δ 3.89	(s)

	δ 7.31	(s)

	Trace amounts	

**Vanillic acid**	δ 6.77/δ 7.22	(d, J = 8.2)/(m)

**Methionine**	δ 2.15	(s)

	δ 2.65	(t, J = 8.0)

A wide range of metabolites is present which includes amino and organic acids (resonances observed in the region of δ 0.80 to 4.00) together with sugars (δ 4.00 to 5.50) and phenolic compounds (δ 5.50 to 8.50).

Malate and succinate contents decreased sharply from *véraison*; the same profile was observed for tartaric acid at δ 4.50 (s), ascorbic acid at δ 4.59 (d, J = 2.0 Hz), and citric acid at δ 2.93 (d, J = 16.0 Hz) with malic and tartaric acids being the most present in grapes (Figure 2, Additional file 2). To confirm if these and other metabolites were present in significantly different amounts during ripening we performed Kruskal-Wallis and Wilcoxon Rank sum tests using spectral intensities at different chemical shifts (*δ *= 0.4-10.0) (see Material and Methods, Additional File 3).

**Figure 3:**
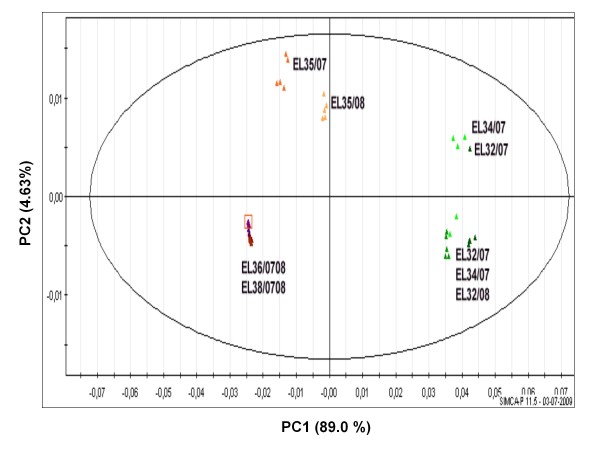
**Score plot of PCA showing metabolic discrimination of developmental stages (EL 32, 34, 35, 36 and 38) corresponding to seasons of 2007 and 2008**. Spectral intensities were scaled to total intensity and reduced to integrated regions of equal width (0.04 ppm). The ellipse represents the Hotelling T2 with 95% confidence in score plots. Sugar region (*δ *3.08-5.48) was removed from the analysis due to bias created by high concentration of sugar compounds.

These spectral intensities were also used for Multivariate Data Analysis using the unsupervised method of Principal component analysis (PCA). A good discrimination was obtained for pre- and post-*véraison *stages when the sugar region (*δ *3.08-5.48) was removed from the analysis (Figure 3). Not surprisingly *véraison *stage (EL 35) appeared clustered apart from all the other stages and showed differences between the two seasons which may be partly due to asynchrony in the onset of ripening known to occur at this stage. Stages EL 35, EL 36 and EL 38 were separated from EL 32 and EL 34 by the first principal component accounting for 89.0% of variance strongly contributed by malate contents. *Véraison *stage (EL 35) was separated from colored berries (EL 36, EL 38) by the second principal component accounting for 4.63% of variance. The stages of EL 36 and EL 38 were clustered together in this analysis.

**Figure 4:**
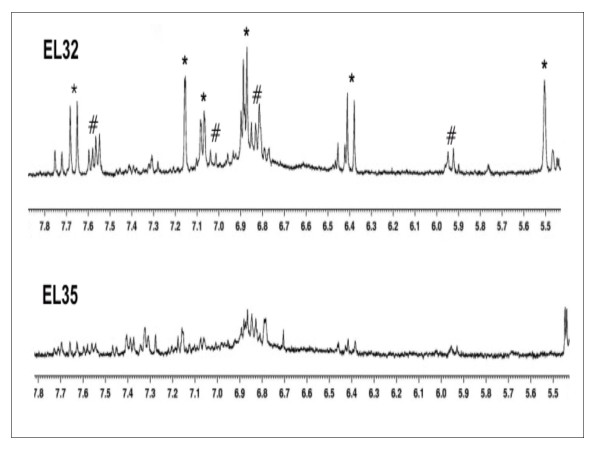
1H NMR spectra at EL 32 and EL 35 showing decrease in contents of *trans*-caftaric acid (*) and *cis*-Coumaroyl derivatives (#) at the onset of ripening.

In order to overcome the congestion of ^1^H NMR spectra mainly due to organic acids and sugars and improve their resolution two-dimensional techniques were carried out. ^1^H NMR together with 2D J-resolved and COSY (correlated spectroscopy) techniques are a reliable methodology for recognition of a broad metabolome, detecting compounds such as amino acids, carbohydrates, organic acids and phenolic compounds. Figure 4 shows ^1^H NMR spectra at EL 32 and EL 35 corresponding partly to the aromatic region (*δ *5.7-9.0), and showing the decrease in *cis*-coumaroyl derivatives and *trans*-caftaric acid (caffeic acid conjugated with tartaric acid) when approaching *véraison*. Identification of these and other compounds was based also on correlation among specific signals given by ^1^H-^1^H correlated spectroscopy (COSY) spectra (Additional File 4) and heteronuclear multiple bonds coherence (HMBC) spectra. While these phenylpropanoids compounds decreased during ripening together with several organic acids and glutamate, contents in vanillic acid, ethyl-beta-glucoside, acetic acid, valine, proline, and γ-amino butyric acid (GABA) were increased in post-*véraison *stages (Additional File 3, for correspondent chemical shifts see Table 1).

**Figure 5:**
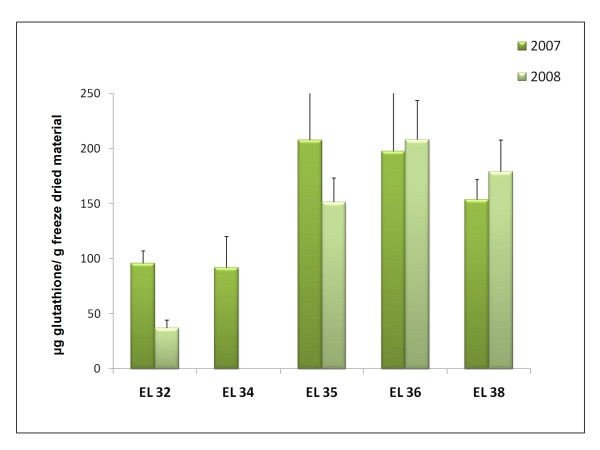
**Total glutathione content expressed in μg per g of freeze dried material**. A spectrofotometric assay was used to measure both oxidized and reduced forms of glutathione [125].

To further characterize the metabolome of grapes during ripening quantification of total glutathione content was performed (Figure 5). This antioxidant compound is a good indicator of oxidative stress present in cells. The results clearly show a significant increase in glutathione at *véraison *and ripe stages comparing to green stages followed by a decrease at harvest stage. Previously, the content in glutathione was shown to increase during grape ripening with 90% being reduced [23] which may indicate an active ascorbate-glutathione cycle.

**Figure 6:**
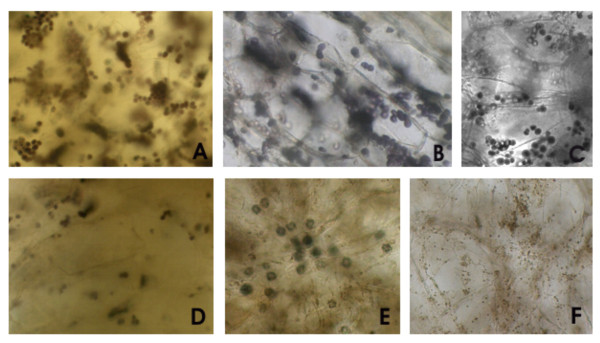
**Starch content evaluated by Lugol staining in pulp cells**. A, B and C correspond to green berries (EL 32, EL 34); D corresponds to *véraison*; E, F correspond to ripe berries (EL 36). In green berries well developed amyloplasts were noticed. In ripe berries (E) druses were observed along with decreased content in starch (E, F).

In order to gather more insights into carbohydrate metabolism, starch content was evaluated in grape sections stained with Lugol solution. In green berries well developed amyloplasts can be observed (Figures 6A, B, C). The number of amyloplasts is reduced at *véraison *(Figure 6D) and decreased content in this polysaccharide was observed during ripening (Figures 6E, F). Interestingly, druses crystals were observed at ripe stages. These structures usually made of calcium oxalate have been previously found in leaves of *Vitis vinifera *and may result from degradation of ascorbic acid in mature grapes [24].

### Microarray and cluster analysis and functional categorization of Unigenes

The mRNA expression profiles of four time points (EL 32, EL 34, EL 35 and EL 36) and two seasons (2007 and 2008) were compared using the Affymetrix GrapeGen^® ^GeneChip genome array containing 23096 probesets corresponding to 18726 unique sequences. Testing was performed using biological triplicates for each time point and datasets from each season were analyzed separately. The quality of the replicates which was checked using Pearson's correlation was very good and ranged between 0.981% and 0.997%. After performing a Bayes t-statistics from the linear models for microarray data (limma) for differential expression analysis [25], P-values were corrected for multiple-testing using the Benjamini-Hochberg's method [26]. The total number of probesets that were differentially expressed (fold change ≥ 1.5 and FDR < 0.05 or fold change ≤ -.1.5 and FDR < 0.05.) was 11759 corresponding to 50.91% of the total probesets represented in the chip. Out of these 7130 probesets were differentially expressed at EL 35 and/or EL 36 in both seasons (Table 2, Additional file 5). This common set of modulated transcripts corresponding to 5877 unigenes indicates the activation of common pathways between years despite the irregular development of Trincadeira grapes. Nevertheless, 2284 and 2345 probesets were differentially expressed only in 2007 and 2008, respectively (Additional file 6). Though the total number of differentially expressed probesets and genes was similar in both seasons in 2008 the amount of genes up-regulated at EL 35 and EL 36 was higher than the amount of genes down-regulated; the opposite was observed in 2007 (Additional file 6). This difference between the two sets likely reflects inter-seasonal biological differences.

**Table 2:** Selection of genes differentially expressed during ripening.

Probe ID	2007 34vs32	2007 35vs32	2007 36vs32	2008 35vs32	2008 36vs32	Unique gene 12× ID	Annotation
**CARBOHYDRATE AND AMINO ACID METABOLISMS**							

VVTU1012_at	.	.	1.77	.	1.61	GSVIVT01033747001	Pyruvate kinase, cytosolic isozyme

VVTU1135_at	3.64	3.82	5.69	2.07	2.77	GSVIVT01012723001	Soluble starch synthase 3, chloroplast precursor

VVTU12019_s_at	.	4.57	5.37	2.3	4.07	GSVIVT01022356001	Aldehyde dehydrogenase

VVTU12208_at	.	-4	-9.68	-2.33	-8.28	GSVIVT01011979001	Phosphoenolpyruvate carboxylase

VVTU12879_at	.	2.73	2.19	2.78	2.37	GSVIVT01024263001	RCP1 (ROOT CAP 1)

VVTU16699_s_at	.	-7.79	-20.35	-2.1	-12.01	GSVIVT01024174001	Fructose-bisphosphate aldolase, chloroplast precursor

VVTU16744_s_at	-1.62	-1.72	-1.82	.	-2.66	GSVIVT01015018001	Sucrose synthase

VVTU17960_s_at	.	.	1.59	.	1.72	GSVIVT01033791001	Fructose-bisphosphate aldolase cytoplasmic isozyme

VVTU1903_at	.	.	-2.26	.	-1.67	GSVIVT01016173001	Malate dehydrogenase [NADP], chloroplast precursor (NADP-MDH)

VVTU1967_s_at	.	1.54	1.94	1.84	2.09	GSVIVT01014206001	Phosphoenolpyruvate carboxylase

VVTU2658_at	.	.	1.5	1.54	1.58	GSVIVT01011700001	Phosphoglucomutase, cytoplasmic

VVTU4210_at	4.86	12.95	23.65	7.73	14.17	GSVIVT01033062001	Alcohol dehydrogenase

VVTU4280_at	3.26	10	13.91	7.05	12.89	GSVIVT01037186001	Sucrose-phosphate synthase 1

VVTU5246_at	.	.	2.14	.	1.86	GSVIVT01006474001	Malate dehydrogenase glyoxysomal

VVTU5612_at	.	-1.85	-4.85	.	-3.3	GSVIVT01013403001	Glyceraldehyde-3-phosphate dehydrogenase B, chloroplast precursor

VVTU7116_at	.	1.82	2.38	1.81	2.19	GSVIVT01008714001	Alpha-amylase/1,4-alpha-D-glucan glucanohydrolase

VVTU8170_at	.	-2.21	-4.09	-1.76	-2.67	GSVIVT01032446001	Glycogen synthase kinase 3 beta

VVTU9506_at	1.54	2.57	1.65	2.66	.	GSVIVT01004839001	Snf1-related protein kinase srk2f

VVTU11854_s_at	.	1.79	1.82	1.51	2.08	GSVIVT01000391001	Glutamate decarboxylase 1 (GAD 1)

VVTU13950_s_at	-1.61	-4.55	-28.07	-2.79	-25.73	GSVIVT01033402001	Glutamate dehydrogenase 1

VVTU14998_at	.	.	4.38	.	2.72	GSVIVT01034731001	Gamma-aminobutyric acid transporter

VVTU22880_s_at	.	1.64	2.02	1.85	3.24	GSVIVT01016467001	Pyrroline-5-carboxylate synthetase

VVTU35297_s_at	.	.	1.55	.	1.7	GSVIVT01036689001	Isocitrate dehydrogenase, chloroplast precursor

VVTU35625_s_at	.	-2.57	-5.34	.	-2.93	GSVIVT01036719001	Succinate-semialdehyde dehydrogenase (SSADH1)

VVTU37879_s_at	.	-2.09	.	.	.	GSVIVT01038714001	GLT1 (NADH-dependent glutamate synthase 1 gene)

VVTU5646_at	.	3.17	3.09	2.18	3.15	GSVIVT01016390001	Proline transporter 1 (ProT1)

VVTU7588_at	.	-2.81	.	-1.73	-1.85	GSVIVT01036483001	Proline oxidase

VVTU977_at	.	.	1.68	.	1.68	GSVIVT01033607001	Cystathionine beta-lyase

**STRESS RESPONSE**							

VVTU12535_s_at	.	.	5.35	.	4.41	GSVIVT01027990001	Glutathione-conjugate transporter (MRP10)

VVTU14104_s_at	.	.	1.73	.	2.13	GSVIVT01033815001	Monodehydroascorbate reductase

VVTU15985_at	.	.	1.59	.	.	GSVIVT01025104001	L-ascorbate peroxidase 1, cytosolic (APX1)

VVTU16784_s_at	.	2.43	3.15	2.94	4.68	GSVIVT01019766001	Phospholipid hydroperoxide glutathione peroxidase

VVTU1974_s_at	.	52.07	88.22	11.76	189.67	GSVIVT01035256001	Glutathione S-transferase 26 GSTF12

VVTU23718_at	.	2.05	.	1.74	2.42	GSVIVT01037479001	L-ascorbate oxidase

VVTU27380_s_at	.	-1.71	-2.42	.	-2.27	GSVIVT01021793001	GDP-mannose 3,5-epimerase 1

VVTU35602_s_at	-1.74	.	-4	.	-1.69	GSVIVT01025551001	L-ascorbate peroxidase 1, cytosolic (APX1)

VVTU38305_s_at	.	3.59	1.63	2.34	2.53	GSVIVT01003998001	Latex cyanogenic beta glucosidase

VVTU40144_at	.	.	.	1.62	.	.	Dehydroascorbate reductase

VVTU40443_s_at	1.94	1.63	1.97	1.83	2.12	GSVIVT01026951001	Beta-cyanoalanine synthase

VVTU4641_at	.	-2.92	-15.77	-1.58	-8.94	GSVIVT01009079001	L-ascorbate peroxidase, chloroplast

VVTU4643_at	.	.	.	-2.03	-2.51	GSVIVT01010646001	L-idonate dehydrogenase

VVTU4990_at	.	2.11	1.97	3.08	2.44	GSVIVT01019757001	Gamma-glutamylcysteine synthetase

VVTU5671_s_at	-2.05	-2.59	-2.86	.	.	GSVIVT01005966001	Dehydroascorbate reductase

VVTU6270_at	.	1.55	2.08	.	1.85	GSVIVT01011626001	Myrosinase precursor

VVTU687_at	.	145.08	240.58	71.81	373.26	GSVIVT01022752001	Anthraniloyal-CoA: methanol anthraniloyal transferase

VVTU7379_at	.	2	1.6	3.1	2.47	GSVIVT01029079001	Glutathione reductase

VVTU8069_at	.	.	-3.45	.	-2.58	GSVIVT01033574001	L-Galactono-1,4-lactone dehydrogenase

**SECONDARY METABOLISM**							

VVTU13083_at	.	-15.92	-10.95	-7.51	-7.09	GSVIVT01006396001	Anthocyanidin reductase

VVTU13266_s_at	-3.1	-5.11	-3.57	-4.5	-2.72	GSVIVT01009731001	Isoflavone reductase protein 4

VVTU13618_x_at	3.48	2.48	.	2.75	.	GSVIVT01028812001	UDP-glucose: anthocyanidin 5,3-O-glucosyltransferase

VVTU13951_at	.	.	3.24	.	1.79	GSVIVT01022411001	Isoflavone reductase

VVTU17578_s_at	.	12.13	14.82	5.19	29.13	GSVIVT01024419001	UDP-glucose:flavonoid 3-O-glucosyltransferase

VVTU20756_at	-3.14	-3.56	-4.09	-2.73	-3.17	GSVIVT01023841001	Dihydroflavonol-4-reductase

VVTU22627_at	.	.	.	.	2.1	GSVIVT01000191001	CYP81E1 Isoflavone 2'-hydroxylase

VVTU39787_s_at	.	-2.43	.	-2.3	4.3	GSVIVT01018781001	Flavonone- 3-hydroxylase

VVTU9453_at	.	.	7.92	1.87	4.75	GSVIVT01019691001	Quercetin 3-O-methyltransferase 1

VVTU9714_at	3.43	4.02	5.02	2.81	3.82	GSVIVT01021355001	Flavonol synthase

VVTU11849_s_at	.	2.15	3.41	1.5	2.64	GSVIVT01026510001	Alcohol dehydrogenase 6

VVTU13316_s_at	.	.	.	-2.21	.	GSVIVT01036331001	(-)-Germacrene D synthase

VVTU21725_at	.	5.59	7.3	7.18	9.32	GSVIVT01026829001	(+)-Neomenthol dehydrogenase

VVTU2626_at	2.55	35.87	19.1	18.1	15.87	GSVIVT01008069001	Isopiperitenol dehydrogenase

VVTU27826_x_at	.	2.5	2.18	1.55	2.01	GSVIVT01003150001	Cinnamyl alcohol dehydrogenase

VVTU33502_at	2.75	.	-2.96	.	-3.52	GSVIVT01032178001	Cinnamyl alcohol dehydrogenase

VVTU37595_s_at	.	2.08	.	1.86	.	GSVIVT01030474001	Hydroperoxide lyase (HPL1)

VVTU4754_at	-1.64	-4.03	-6.42	-4.25	-7.87	GSVIVT01008854001	Caffeic acid methyltransferase

VVTU8254_at	.	4.4	7.29	2.5	2.95	GSVIVT01036862001	9-*cis*-epoxycarotenoid dioxygenase

**METABOLISM AND SIGNALING OF GROWTH REGULATORS**							

VVTU1335_at	1.65	-6.21	-7.81	-3.38	-6.13	GSVIVT01000176001	Indole-3-acetic acid-amido synthetase GH3.2

VVTU16083_at	.	.	-2.96	.	-2.18	GSVIVT01030905001	Auxin efflux carrier family

VVTU16124_at	.	.	-2.05	-1.82	-2.87	GSVIVT01031663001	PIN1

VVTU1813_at	-3.17	-12.35	-48.38	-4.69	-33.36	GSVIVT01017046001	IAA9

VVTU18738_s_at	.	14.93	37.41	22.78	87.35	GSVIVT01038622001	Auxin-responsive SAUR29

VVTU2445_s_at	-2.2	-13.15	-17.4	-6.43	-9.33	GSVIVT01015350001	Auxin-responsive protein IAA27

VVTU2614_s_at	.	2.08	1.68	1.5	1.79	GSVIVT01033011001	Transport inhibitor response 1 protein

VVTU3361_at	3.34	9.44	9.88	6.46	9.06	GSVIVT01017158001	IAA19

VVTU35572_s_at	2.81	2.25	4.41	3.04	8.58	GSVIVT01020159001	IAA-amino acid hydrolase 1 (ILR1)

VVTU3560_at	-1.83	.	2.93	.	3.86	GSVIVT01037892001	Indole-3-acetic acid-amido synthetase GH3.8

VVTU35909_s_at	.	-2.42	.	-2.25	-1.69	GSVIVT01026429001	Auxin Efflux Carrier

VVTU38338_x_at	-1.59	-11.61	-14.02	-9.85	-22.64	GSVIVT01024135001	Auxin-responsive SAUR31

VVTU7869_at	-5.63	-6.03	-10.54	-6.2	-4.14	GSVIVT01010995001	Transport inhibitor response 1

VVTU12042_at	1.76	.	.	.	.	GSVIVT01005455001	1-Aminocyclopropane-1-carboxylate synthase

VVTU12870_s_at	.	.	1.83	.	2.14	GSVIVT01025105001	MAPK (MPK3)

VVTU13344_at	.	-1.68	-2.66	.	-4.88	GSVIVT01006065001	1-Aminocyclopropane-1-carboxylate oxidase 1

VVTU1588_at	.	.	1.62	.	1.99	GSVIVT01038085001	Ethylene receptor 1 (ETR1)

VVTU18607_s_at	3.66	29.17	28.93	14.04	40.01	GSVIVT01035911001	Ethylene-responsive transcription factor ERF003

VVTU19389_s_at	.	.	1.73	.	2.05	GSVIVT01036213001	Ethylene receptor (EIN4)

VVTU2683_s_at	.	-1.8	.	-2.23	.	GSVIVT01035856001	EIN3-binding F-box protein 2

VVTU35437_at	.	-1.58	-5.17	2.26	2.62	.	Ethylene-responsive transcription factor ERF105

VVTU5165_at	.	-2.11	-1.79	.	-1.57	GSVIVT01008900001	1-Aminocyclopropane-1-carboxylate synthase

VVTU5909_at	.	1.9	1.59	1.87	1.62	GSVIVT01011670001	1-Aminocyclopropane-1-carboxylate oxidase

VVTU8172_at	.	.	2.31	2.76	12.06	GSVIVT01004798001	Ethylene responsive element binding factor 1

VVTU8555_at	.	-3.58	-4.58	-2.09	-5.28	GSVIVT01037473001	Ethylene-insensitive 3 (EIN3)

VVTU11913_at	-2.04	-5.96	-11.68	-3.88	-16.02	GSVIVT01018733001	Jasmonate O-methyltransferase

VVTU16057_at	.	9.26	10.63	5.74	7.16	GSVIVT01009616001	Allene oxide synthase

VVTU1657_s_at	-2.04	.	-2.45	-2.41	-2.7	GSVIVT01005061001	Methyl jasmonate esterase

VVTU16654_at	1.58	2.35	1.62	1.89	1.77	GSVIVT01031706001	IMP dehydrogenase

VVTU17030_s_at	.	-11.17	-8.28	.	-4.33	GSVIVT01025923001	12-Oxophytodienoate reductase 2

VVTU23697_at	.	1.6	2.16	1.99	2.72	GSVIVT01016368001	Coronatine-insensitive protein 1

VVTU3032_at	.	.	.	.	1.67	GSVIVT01027057001	JAR1-like protein

VVTU34392_at	2.43	.	.	.	.	GSVIVT01013156001	MYC jasmonic acid 3

VVTU35149_at	.	-1.72	.	-1.55	.	GSVIVT01024198001	Enhanced disease susceptibility 5 EDS5

VVTU39811_s_at	.	2.76	50.75	.	38.44	GSVIVT01021514001	Jasmonate ZIM domain-containing protein 8

VVTU4273_s_at	-1.53	.	-1.58	.	-1.98	GSVIVT01008453001	Jasmonate ZIM domain-containing protein 3

VVTU7003_at	-2.47	-12.82	-13.47	-6.21	-13.03	GSVIVT01036445001	Allene oxide cyclase

VVTU7560_at	.	.	2.04	1.65	2.99	GSVIVT01015181001	Regulatory protein NPR1 (Nonexpresser of PR genes 1)

VVTU1269_s_at	.	1.52	.	1.56	.	GSVIVT01020222001	Spermidine synthase

VVTU12839_at	.	1.64	2.39	3.44	4.27	GSVIVT01024167001	Arginine decarboxylase (Fragment)

VVTU12964_s_at	1.88	.	1.81	1.8	2.66	.	S-Adenosylmethionine decarboxylase proenzyme

VVTU37047_at	.	.	1.87	.	3.11	GSVIVT01007669001	Copper amine oxidase

VVTU5224_at	.	.	2.17	.	1.51	GSVIVT01028700001	Spermine synthase

VVTU5226_at	.	2.19	1.76	1.69	2.42	GSVIVT01020812001	Amine oxidase

VVTU6472_at	-2.27	2.07	.	1.86	2.07	GSVIVT01004079001	Copper amine oxidase

VVTU8738_s_at	.	2.3	2.17	.	.	GSVIVT01033651001	S-Adenosylmethionine synthetase

VVTU12347_s_at	.	.	.	2.03	.	GSVIVT01009074001	SnRK2-8

VVTU19049_s_at	.	.	2.01	.	1.95	GSVIVT01037491001	UBP1 interacting protein 2a (UBA2a)

VVTU22232_at	.	-1.91	-2.11	.	.	GSVIVT01003554001	Snf1 protein kinase 2-3 akip ost1

VVTU28731_s_at	2.01	4.9	4.9	4.67	3.13	GSVIVT01015308001	ABI1 (ABA insensitive 1)

VVTU14956_at	2.22	1.89	1.75	1.8	1.55	GSVIVT01008164001	BIM1 (BES1-interacting Myc-like protein 1)

VVTU24849_at	.	-1.92	-1.91	-3.07	-4.02	GSVIVT01017237001	CYP734A7 castasterone 26-hydroxylase

VVTU4905_s_at	.	.	-2.3	-2.41	-2.1	.	Brassinosteroid-responsive ring-H2 (BRH1)

VVTU647_at	.	-12.51	-17.26	-3.26	-21.67	GSVIVT01036558001	Brassinosteroid-6-oxidase

VVTU20270_s_at	-1.93	.	3.68	.	7.79	GSVIVT01033610001	ARR3 typeA

VVTU28950_s_at	.	-4.38	-11.11	-1.85	-3.95	GSVIVT01004944001	Cytokinin-repressed protein CR9

VVTU31519_s_at	3.4	.	.	1.6	.	GSVIVT01027443001	Pseudo-response regulator 9 (APRR9)

VVTU9094_s_at	.	-5.82	-7.62	-5.17	-14.3	GSVIVT01035468001	Cytokinin dehydrogenase 7

VVTU9297_at	-2.85	-8.33	-6.37	-3.83	-3.2	GSVIVT01007835001	ARR6 typeA

VVTU9337_at	2.81	2.61	4.69	1.92	6.66	GSVIVT01035051001	ARR1 typeB

VVTU13918_at	.	10.7	40.6	27.15	38.26	GSVIVT01031830001	Gibberellin 20 oxidase 2

VVTU15195_at	.	-1.59	4.64	.	2.89	GSVIVT01022014001	Gibberellin receptor GID1L1

VVTU1752_at	3.79	12.25	12.84	4.95	4.98	GSVIVT01011037001	Gibberellin receptor GID1L2

VVTU7332_at	-2.92	-6.26	-6.69	-4.5	-7.87	GSVIVT01009099001	Gibberellin 20 oxidase 2

VVTU8591_at	.	-4.73	-4.46	-4.09	-5.78	GSVIVT01034945001	Gibberellin 2-oxidase

**SIGNAL TRANSDUCTION**							

VVTU11835_at	.	1.55	.	1.76	1.62	GSVIVT01018839001	MADS box transcription factor TM6 (TM6) APETALA3

VVTU17564_s_at	.	8.95	11.56	4.78	18.34	GSVIVT01022664001	Myb VvMYBA3 [Vitis vinifera]

VVTU18199_s_at	.	.	1.62	1.76	1.85	GSVIVT01033067001	SEPALLATA3

VVTU2522_at	.	1.56	2.63	.	3.24	GSVIVT01016175001	NAC domain-containing protein 78

VVTU27392_s_at	.	3.53	4.76	2.16	3.94	.	Scarecrow-like transcription factor 8 (SCL8)

VVTU3046_s_at	.	-6.64	-5.33	-2.63	-3.25	GSVIVT01027182001	MYBPA1 protein [Vitis vinifera]

VVTU3183_at	.	2.05	.	1.54	.	GSVIVT01024921001	Zinc finger (C3HC4-type RING finger)

VVTU3258_at	-1.75	-126.42	-210.41	-28.95	-221.25	GSVIVT01037819001	LIM domain protein WLIM1

VVTU37071_at	.	.	.	.	2.06	GSVIVT01034155001	Scarecrow-like transcription factor 9 (SCL9)

VVTU40803_s_at	2.35	4.93	9.8	1.54	6.18	GSVIVT01034968001	WRKY DNA-binding protein 48

VVTU9543_at	.	2.12	8.24	1.77	8.89	GSVIVT01022269001	Myb TKI1 (TSL-KINASE INTERACTING PROTEIN 1)

VVTU11578_at	1.6	12.25	4.66	2.82	1.77	GSVIVT01008070001	Receptor protein kinase

VVTU11917_at	2.55	1.53	.	2.18	.	GSVIVT01019481001	BZip transcription factor G-BOX BINDING FACTOR 3

VVTU13369_at	.	1.85	.	1.97	.	GSVIVT01017690001	CBL-interacting protein kinase 1 (CIPK1)

VVTU2538_at	.	1.68	.	1.83	1.5	GSVIVT01033306001	CALCIUM-DEPENDENT PROTEIN KINASE 32 CPK32

VVTU26057_at	.	5.13	12.44	8.86	17.28	GSVIVT01016073001	STE20/SPS1 proline-alanine-rich protein kinase

VVTU27362_at	1.53	1.74	2.13	2.55	5.29	GSVIVT01034540001	bZIP transcription factor

VVTU3691_at	.	3.73	.	1.6	.	GSVIVT01010053001	Dof zinc finger protein DOF3.5

VVTU38545_at	.	1.76	3.18	.	3.59	GSVIVT01008327001	Wall-associated kinase 4

VVTU5563_at	.	2.6	3.52	2.09	2.53	GSVIVT01034897001	VirE2-interacting protein (VIP1)

VVTU8084_at	.	.	2.1	.	2.62	GSVIVT01036465001	Receptor protein kinase PERK1

VVTU9535_at	.	2.78	4.54	3.85	4.3	GSVIVT01002864001	Receptor protein kinase PERK1

VVTU9861_at	.	1.92	2.09	1.85	2.19	.	Wall-associated kinase

**LIGHT SIGNALING, CIRCADIAN CLOCK, EPIGENETIC FACTORS AND TRANSPOSONS**							

VVTU22197_at	.	.	1.95	1.52	1.79	GSVIVT01007965001	Timing of CAB expression 1 protein

VVTU2284_at	.	1.76	4.05	.	3.36	GSVIVT01035337001	Early flowering 3

VVTU2454_s_at	2.4	.	1.77	3.04	2.15	GSVIVT01001405001	Gigantea protein

VVTU3515_s_at	-1.65	-1.58	-1.74	-1.89	-2.32	GSVIVT01027456001	Myb CCA1 (Circadian Clock Associated 1)

VVTU40867_x_at	.	2.19	.	2.47	2.44	GSVIVT01018044001	ELIP1 (Early Light-Inducible Protein)

VVTU5883_at	.	-1.59	.	2.17	2.7	GSVIVT01030081001	Phytochrome defective C (PHYC)

VVTU10989_at	-2.75	1.77	.	-2.1	1.55	GSVIVT01033746001	Retrotransposon protein, Ty1-copia subclass

VVTU11309_at	.	-1.72	-2.05	.	.	GSVIVT01032746001	Chromatin remodeling 42

VVTU12696_at	.	2.96	2.08	2.38	1.99	GSVIVT01033971001	Transposon protein, CACTA, En/Spm sub-class

VVTU15783_at	.	.	2.05	.	2.48	.	Retrotransposon protein, unclassified

VVTU2258_at	2.29	7.14	2.59	1.77	2.61	GSVIVT01010060001	DNA-3-methyladenine glycosidase I

VVTU32711_at	.	.	.	2.38	.	GSVIVT01017791001	Chromatin-remodeling protein 11

VVTU3690_at	1.53	2.15	3.56	2.05	3.61	GSVIVT01007671001	Histone deacetylase HDA6

VVTU38460_at	.	.	.	2.68	2.01	GSVIVT01026952001	ATBRM/CHR2 (Arabidopsis thaliana brahma)

VVTU5491_at	.	.	2.27	.	2.08	.	Transposase

VVTU5815_at	.	.	1.64	.	1.68	GSVIVT01020136001	Histone deacetylase complex, SIN3 component

VVTU6149_s_at	.	2.09	-1.85	1.54	.	GSVIVT01033869001	Transposon protein, Mutator sub-class

VVTU8524_at	-1.64	-1.75	-2.04	.	-1.57	.	Cytosine methyltransferase (DRM2)

VVTU8618_at	.	.	2.12	.	2.34	GSVIVT01007544001	Histone acetyltransferase ELP3

VVTU87_at	.	.	-2.41	.	-1.74	GSVIVT01007870001	Histone deacetylase HDA05

The selection considered a fold change ≥ 1.5 and FDR < 0.05 or fold change ≤ -.1.5 and FDR < 0.05).

**Figure 7:**
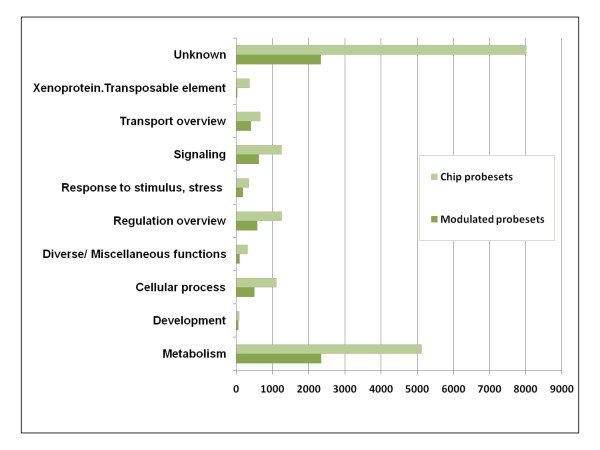
**Functional categories distribution in the core set of the 7130 modulated genes and in the entire GrapeGen Chip^®^**.

Functional annotations have been assigned to the majority of probesets though 32.79% of the core set of 7130 genes had matches to genes with unknown functions (Figure 7). The assignment to functional categories was performed assigning each gene to a category according to its putative molecular function. Nine categories beside the genes with unknown function were represented during berry development in the regulated gene core set. These were "metabolism", "development", "cellular process", "diverse/miscellaneous functions", "regulation overview", "response to stimulus, stress", "signaling", "transport overview", and "xenoprotein, transposable element". The number of modulated probesets related to metabolism was similar to the number of those having unknown function (2343 and 2338, respectively). Two functional categories were not represented in the gene core set but in the chip namely "Cellular response overview", and "Xenoprotein, viral protein". This later one was represented in the set of genes modulated in only one season (Additional file 6).

**Figure 8:**
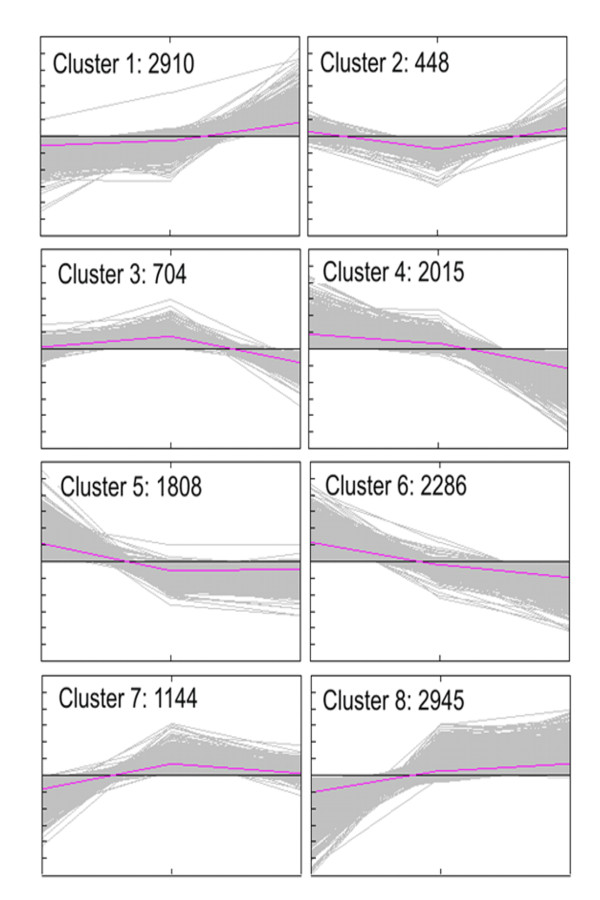
**Clustering of the expression profiles of the core set of the 7130 modulated genes across three developmental stages of grape ripening (EL 32, EL 35 and EL 36)**. Clustering was performed using *k*-means statistics and the number of genes in each cluster (eight) is shown.

Cluster analysis of the gene core set was based on the *k*-means method using Pearson's correlation distance calculated on the gene expression profiles obtained for EL 32, EL 35 and EL 36 in both years. Probesets were clustered into eight groups representing the minimum number of profiles that can be obtained with 3 time points (Figure 8).

We did not observe a good agreement between clustering in the gene core set from the 7130 probesets that were differentially expressed at EL 35 and/or EL 36 in 2007 and 2008 since only 3451 of the transcripts (48,40%) fell in the same cluster in both seasons (Additional file 5). Among the 3451 probesets that showed a conserved profile in the two seasons, we identified clusters 1 and 8 as the most populated ones. These clusters correspond to transcripts that were positively modulated after *véraison *(885) and at *véraison *and ripe stage (786), respectively. Cluster 7 (250) and cluster 3 (147) indicate genes showing a peak of expression at *véraison *with the latter representing genes also down-regulated at EL 36. Cluster 5 (400) and cluster 6 (467) represent genes repressed at EL 35 and EL 36, though the latter represent genes showing also a gradual decrease in expression from EL 35 to EL 36. Cluster 4 (445) accounts for genes being repressed at EL 36 and cluster 2 (71) represent genes showing the lowest level of expression at *véraison*.

Clusters 1 and 8 shows enrichment in genes annotated as involved in regulation of gene expression indicating the complexity of transcriptional regulation during berry ripening. On the other hand, clusters 4 and 6 indicate that following *véraison *there is an increase in genes down-regulated involved in transport mechanisms. When we compare clusters 2 and 7 we can conclude that in the latter there are less genes involved in primary metabolism and transport overview, and more genes involved in secondary metabolism and hormone signaling (Additional file 5). The results indicate that *véraison *is a stage of active metabolism of aminoacid, carbohydrate and lipids together with their transport as well as water transport mediated by aquaporins.

Clusters 5 and 6 have increased number of genes annotated as involved in cellular component organization and biogenesis due to high cellular pre- *véraison *activity and suggesting cellular reprogramming at the onset of *véraison*.

### Analysis of gene expression during grape berry ripening

#### Carbohydrate metabolism

Berries start to accumulate after *véraison *the carbohydrates produced during photosynthesis and imported from the leaves.

In Trincadeira berries sucrose concentrations increased throughout berry development though glucose content was higher (Figure 2). This is in contrast with the results obtained for Cabernet Sauvignon during which sucrose content remained relatively constant [15]. Transcript abundance of genes encoding enzymes involved in sucrose biosynthesis was higher at EL 36 (Figure 2, Table 2), namely sucrose-phosphate synthase 1 (VVTU4280_at, cluster 8) and sucrose phosphatase (VVTU21174_s_at, cluster 8). This last enzyme catalyzes the final step in the pathway of sucrose synthesis. Other authors [16] also mentioned up-regulation of genes coding for sucrose-phosphate synthase and sucrose-6-phosphate phosphatase in ripe Pinot Noir berries but did not quantify sucrose.

An interesting feature is that both studies on Cabernet Sauvignon and Pinot Noir showed up-regulation of genes encoding sucrose synthase whereas in Trincadeira this gene is down-regulated (VVTU16744_s_at) consistent with an increase in sucrose levels.

Plastids of ripening berries have an active and complex starch metabolism. Lugol staining showed decreased levels of starch in mesocarp cells at EL 35 and EL 36 as previously described [15] and consistent with increased transcript abundance of Unigenes involved in starch degradation and coding for alpha-glucan phosphorylase, H isozyme (VVTU6785_s_at, cluster 7), beta-amylase (VVTU15830_s_at), isoamylase isoform 3 (VVTU5803_s_at, cluster 8), and alpha-amylase (VVTU7116_at, cluster 8). Moreover, transcripts encoding fructokinases (VVTU2588_s_at, VVTU4521_at), which catalyzes the formation of fructose-6-phosphate and may regulate starch formation, were down-regulated. Alpha-amylase is an enzyme which aids in the breakdown of starch to maltose, a compound that can act as an osmoprotectant [27]. It should be noted the up-regulation at EL 35 and EL 36 of a RCP1 (ROOT CAP 1) gene (VVTU12879_at, cluster 7) putatively coding for a Maltose transporter based on homology with ESTs (Additional files 5, 6).

Though starch content decreases in berries at EL 35 and EL 36 (Figure 6), genes putatively involved in synthesis of starch such as coding for Starch synthase 1 and 3, chloroplast precursors (VVTU23087_s_at, cluster 8, VVTU1135_at, cluster 8) and ADP-glucose pyrophosphorylase large subunit 2 (VVTU17473_at, cluster 8) were up-regulated during ripening while other genes putatively coding for isoenzymes were down-regulated (VVTU11416_at, cluster 6; VVTU12614_at, cluster 3, Additional file 5). The up-regulation of a gene coding for starch synthase was also observed for ripening of Cabernet Sauvigon grapes [15]. In fact, the control of activity of starch synthesis and degradation enzymes is complex in storage organs such as fruits. Different starch degradation pathways may be specific to early development and not active in late development [28].

Sucrose Non Fermenting 1 (SNF1)-related kinase and hexokinase are involved in sugar signaling pathways modulating post-translational redox activation of ADP-Glc pyrophosphorylase [29]. We report here the putative involvement of this sugar-inducible protein kinase in the onset of grape ripening. In fact, a gene coding for a SNF1-RELATED PROTEIN KINASE SRK2F (VVTU9506_at, cluster 7) putatively involved in hyperosmotic response [30] was up-regulated only at EL 35 (*véraison*). In plants, SNF1 [sucrose non-fermenting 1]-related kinase 1 seems to have important roles in controlling metabolic homeostasis and stress signalling [31]. Recently, a Glycogen Synthase Kinase3 protein kinase, VvSK1 (Sugar-Inducible Protein Kinase), was shown to regulate sugar accumulation in grapevine cell suspension [32]. In the case of Trincadeira grape ripening, a gene coding for a glycogen synthase kinase 3 beta (VVTU8170_at, cluster 6) was down-regulated at EL 35 and EL 36 which may be due to cultivar specificities.

Plastid glycolysis seems to be inhibited at the onset and following *véraison *as several genes coding for plastidial phosphoglycerate kinase (VVTU1271_at, cluster 6), glyceraldehyde-3-phosphate dehydrogenase A and B (VVTU17859_s_at, VVTU5612_at, cluster 4), and fructose bisphosphate aldolase (VVTU16699_s_at, VVTU1150_s_at) are down-regulated at these stages. On the other hand, cytoplasmic glycolysis seems to be activated. In fact, genes coding for cytosolic Phosphoglycerate kinase (VVTU18434_s_at, cluster 1), fructose-bisphosphate aldolase cytoplasmic isozyme (VVTU17960_s_at, cluster 1), cytoplasmic phosphoglucomutase (VVTU2658_at, cluster 8) and pyruvate kinase, cytosolic isozyme (VVTU1012_at, cluster 1) are up-regulated.

In the past, it was reported for whole berry analysis that glycolysis is down-regulated after *véraison *[17]. Other transcriptomic and proteomic analysis conducted on the whole berry or only skin showed that several glycolytic enzymes increased during ripening [13,18]. Although different berry tissues may have different trends of glycolysis [18], we highlight here that cellular compartmentation should be taken into account, an issue that up to our knowledge has not been previously adressed.

This increase in the rate of cytoplasmic glycolysis due to an excess of sugars leads to an increase in pyruvate that may trigger aerobic fermentative metabolism [33]. In fact, the production of ethanol by pyruvate decarboxylase and alcohol dehydrogenase may occur in ripening fruit (reviewed by [34]). Pilati et al. [16] observed up-regulation of genes coding for alcohol dehydrogenase and aldehyde dehydrogenase which may be indicative of a shift to an aerobic fermentative metabolism during ripening [35].

We observed that genes coding for an Alcohol dehydrogenase 6 (VVTU6090_s_at) and Alcohol dehydrogenase (VVTU4210_at, cluster 8) were up-regulated at EL 35 and 36. Metabolic profiling indicates for these samples the presence of 1-O-ethyl-beta-glucoside which may derive from the transfer of the glucosyl moiety from a group of phenolic beta-glucosides to ethanol; this latter compound is known to control cytosolic acidity in ripe grapes [36]. This data may indicate that aerobic fermentation is occurring during ripening of Trincadeira grapes. Moreover, a gene coding for aldehyde dehydrogenase (VVTU12019_s_at, cluster 8) was up-regulated at EL 35 and even more at EL36. Giribaldi and co-workers [17] also observed in proteomic studies an increase in presence of aldehyde dehydrogenase isoforms during grape ripening, and related it with recycling of ethanol after *véraison *[13].

Organic acids such as malic and tartaric acids are well known for their contribution to wine taste. In the cytoplasm, malate can be produced from PEP produced in glycolysis through the activities of phosphoenolpyruvate carboxylase (PEPC) and malate dehydrogenase. Though one Unigene coding for a PEPC was up-regulated at ripe stage (VVTU1967_s_at, cluster 8), two genes were down-regulated (VVTU12208_at, VVTU19092_at) at *véraison *and ripe stages in agreement with a decrease in malate (Figure 2). Since malate dehydrogenase catalyzes a reversible reaction between oxaloacetate and malate, malate dehydrogenase may be involved in malate synthesis, which occurs mainly pre-*véraison *and malate degradation at post-*véraison*. Several isoforms of malate dehydrogenase operating in different cellular compartments may control the net content in malate. Two malate dehydrogenase isoenzymes, one glyoxysomal, were up-regulated (VVTU2535_at, cluster 8; VVTU5246_at, cluster 1) whereas two isoenzymes one plastidial and one glyoxysomal were down-regulated during ripening (VVTU4095_at, VVTU1903_at).

Malic enzyme catalyzes the reversible conversion between malate and pyruvate. Two genes coding for NADP-dependent malic enzyme were either up-regulated at EL 35, and EL36 in 2008 (VVTU18630_at), or in 2007 (VVTU35950_at) (Additional files 5, 6). Environmental factors such as temperature may activate particular pathways of malate degradation but it is also possible that different tissues behave differently. Anyhow, the regulation of malate concentrations in berries is very complex [15]. Recently, it has been showed that Trincadeira presents higher concentrations of malate than other Portuguese cultivars [20] but more research is needed to gather insights into the carbohydrate metabolism of this particular variety.

#### Amino acid metabolism

Amino acids such as proline play a role in wine taste by interfering with the sensation of acidity due to their buffering capacity [37]. During ripening we observed an increase in most amino acids but not for glutamate (Additional file 3). In fact, this amino acid decreases during ripening and a gene coding for Glutamate dehydrogenase 1 (VVTU13950_s_at, cluster 4) is down-regulated especially at EL 36.

Interestingly one gene coding for GLT1 (NADH-dependent glutamate synthase 1) (VVTU37879_s_at) was down-regulated at *véraison *in 2007 but not in 2008, accounting for differences in nitrogen metabolism between seasons. This is further supported by the fact that a gene coding for nitrate reductase is down-regulated during ripening but only in 2008 (VVTU9432_at, Additional file 6).

Glutamate may be catabolized through glutamate decarboxylase, into γ-aminobutyric acid (GABA), a metabolite that increases during ripening. A gene coding for a glutamate decarboxylase (VVTU11854_s_at, cluster 8) was up-regulated at EL 35 and EL 36.

Interestingly, an increase in the transcript abundance of a gene coding for a gamma-aminobutyric acid transporter (VVTU14998_a, cluster 1) was noticed at ripe stage (EL36) when there is increased oxidative stress and sugar accumulation.

**Figure 9:**
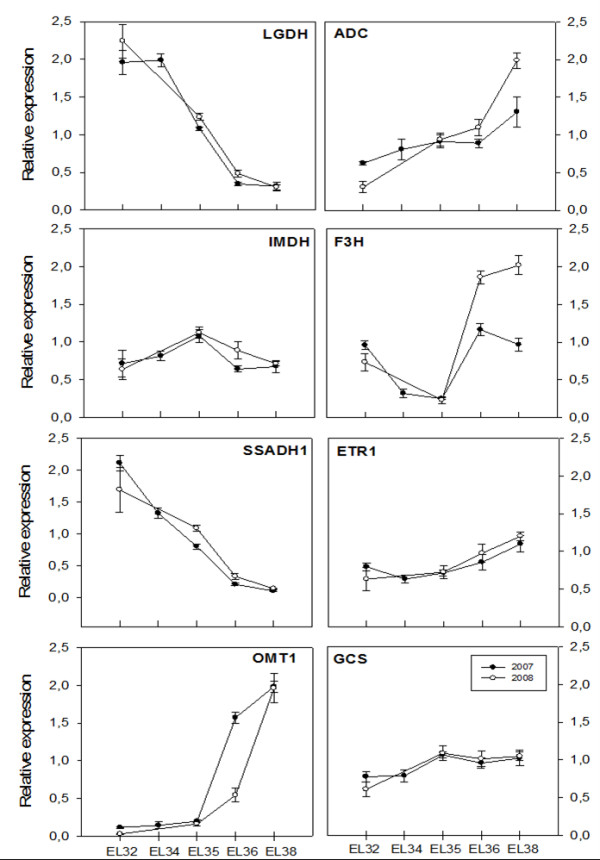
**Real time RT-PCR validation of the expression profiles of eight genes in the two seasons under analysis**. Data are reported as means ± SE of three technical and two biological replicates. Transcript levels were calculated using the standard curve method and normalized against grapevine actin gene (VVTU17999_s_at) used as reference control. VVTU8069_at: L-galactono-1,4-lactone dehydrogenase (LGDH), VVTU12839_at: Arginine decarboxylase (ADC), VVTU16654_at: IMP dehydrogenase (IMDDH), VVTU39787_s_at: Flavonone- 3-hydroxylase (F3H), VVTU35625_s_at: Succinic semialdehyde dehydrogenase (SSADH1), VVTU1588_at: Ethylene receptor 1 (ETR1), VVTU9453: Quercetin 3-O-methyltransferase 1 (OMT1), VVTU4990_at: Gamma-glutamylcysteine synthetase (GCS).

During ripening a transcript encoding a Succinic semialdehyde dehydrogenase (SSADH1; VVTU35625_s_at) putatively involved in GABA degradation is down-regulated in both seasons as obtained by both microarray and qPCR analysis (Figures 2, 9, Table 2). This enzyme participates in the GABA shunt from which results succinate which content also decreases at ripe stages. In citrus fruit, also a non-climacteric fruit, the GABA shunt was suggested to play an important role in reduction of citrate and cytoplasmatic activity during ripening [38]. However, our results don't suggest this probably because malate is the organic acid accounting for most of titrable acidity instead of citrate which is the case of citrus. In this fruit, alternative citrate breakdown catalyzed by ATP citrate lyase was ruled out since the corresponding gene was clearly down-regulated [38]. On the contrary, in Trincadeira grapes this gene was either not differentially expressed or up-regulated with a low fold change (not shown).

The observed decreased levels in citrate following *véraison *should be also due to the action of NADP isocitrate dehydrogenase involved in conversion of isocitrate into 2-oxogutarate. A gene coding for an isocitrate dehydrogenase, chloroplast precursor (VVTU35297_s_at, cluster 8) and a gene coding for a Isocitrate dehydrogenase (NAD+) precursor (VVTU4698_at) were both up-regulated at EL 36.

Nevertheless glutamate may be partly consumed by the GABA shunt since during ripening there are increased levels of GABA. Alternatively, may be consumed for proline synthesis since the levels of this amino acid strongly increased during ripening and a gene encoding pyrroline-5-carboxylate synthetase (VVTU22880_s_at, cluster 8) involved in proline synthesis was up-regulated. The same increase in proline and proline biosynthetic gene was reported for ripening of Cabernet Sauvignon grapes [15]. This amino acid may be playing a role as osmoprotectant during ripening stages [39,40].

In accordance, a gene coding for a proline oxidase was down-regulated during ripening (VVTU7588_at, cluster 5). Interestingly, a gene coding for proline transporter 1 (ProT1, VVTU5646_at, cluster 8) was up-regulated at EL 35 and EL 36.

A good correlation was obtained with a transcript profile for a gene coding for Cystathionine beta-lyase (VVTU977_at) putatively involved in methionine biosynthesis and its increased content at EL 36 (Table 1, Additional file 3). It is likely that it plays a role in providing a pool of S-Adenosyl methionine for polyamines' biosynthesis as it will be discussed in another section of this paper. The pool of these growth regulators should also control arginine metabolism. Though for most amino acids a good correlation was obtained for their content and the genes involved in their biosynthesis, this was not the case for this amino acid. In fact, arginine levels increase at ripe and mainly at harvest stages. However, a gene coding for arginine decarboxylase (VVTU12839_at, cluster 8 - Arginine decarboxylase (Fragment) involved in arginine catabolism increases at EL35 and EL36 (Table 2, Figure 9). Moreover, a gene coding for Glutamate N-acetyltransferase (VVTU22296_s_at) involved in synthesis of ornithine and arginine was down-regulated at EL36.

#### Stress response

Glutathione transferases are known to be up-regulated in many plants in response to a range of stress conditions [41]. We observed a transcript encoding a *Vitis vinifera *glutathione S-transferase 26 (GSTF12) (VVTU1974_s_at, cluster 8) that displayed an 88 and 190-fold increase in abundance at EL 36 in 2007 and 2008 respectively, and may be involved in anthocyanin sequestration in vacuoles [41]. Interestingly, a gene coding for a glutathione-conjugate transporter (MRP10; VVTU12535_s_at, cluster 1) was up-regulated at EL36 in both seasons. To our knowledge this transporter has not been previously described in the context of grape ripening.

Pilati and co-workers [16] have reported the occurrence of an oxidative stress burst during grape ripening as it has been reported for other climacteric and non-climacteric fruits namely tomato [42], strawberry [43], pineapple [44] and pepper [45]. The occurrence of oxidative stress during grape berry development has been rather controversial since at the transcriptional level many typical oxidative stress markers seemed absent or negatively regulated [13]. It should be also taken into account that grapes accumulate many phenylpropanoids that can play an antioxidant role. For instance, procyanidin, catechin, epicatechin and gallic acid scavenged a stable free radical much more efectively than the antioxidant ascorbic acid [46].

Our results support the results of Pilati and co-workers [16] since like berry H_2_O_2_, glutathione increased significantly at EL 35 reaching a maximum two weeks after and decreasing at harvest. A gene coding for Gamma-glutamylcysteine synthetase (VVTU4990_at, cluster 7) involved in glutathione biosynthesis was also up-regulated during ripening in both 2007 and 2008 (Table 2, Figure 9). Further studies are required to figure out the role played by oxidative stress in ripening. An increase in the levels of glutathione was previously observed during ripening of Koshu and Cabernet Sauvignon grapes [23]. The activities of catalase, nonspecific peroxidase, and ascorbate peroxidase were undetectable in these grapes during ripening, in contrast with the activities of glutathione reductase, dehydroascorbate reductase, and glutathione peroxidase. In our study, several genes coding for isoforms of catalase, peroxidase, superoxide dismutase, glutathione peroxidase, phospholipid hydroperoxide glutathione peroxidase, and ascorbate peroxidase were up and down-regulated during ripening though in certain cases only in one a season eventually due to tissue specificities and/or weather conditions (Table 2, Additional file 6).

Much evidence has been gathered pointing to a pivotal role for the ascorbate-glutathione cycle in scavenging reactive oxygen species. Its activity relies on the sequential oxidation and re-reduction of ascorbate and glutathione. We found genes coding for enzymes of the cycle that were up-regulated during ripening (VVTU7379_at, cluster 7 - Glutathione reductase, VVTU14104_s_at, cluster 1 - monodehydroascorbate reductase, and VVTU13460_at- L-ascorbate peroxidase 1, cytosolic APX1) except for dehydroascorbate reductase (VVTU5671_s_at - dehydroascorbate reductase) which was down-regulated but only in 2007 (Table 2, Additional file 6), and reduces dehydroascorbate to ascorbate using reduced glutathione as the reducing agent. One gene though coding for a dehydroascorbate reductase (VVTU40144_at) increased its transcript abundance by 1.62 fold at EL 35 but only in 2008.

This data together with the fact that ascorbate levels decrease and glutathione levels increase make it difficult to ascertain an important role for this cycle during ripening as it has been described for tomato [42]. Moreover, this cycle operates in compartments such as chloroplasts, mitochondria, and peroxisomes and tissue specific activity may be expected. For instance, it has been reported that the concentrations of ascorbate and glutathione in apple epidermis were higher than in the underlying mesocarp [47]. In Trincadeira grapes we found a general tendency for these genes to display higher transcript abundance in 2008 (Additional file 6).

We found a good correlation between the decrease in ascorbate levels (Additional file 3) and the expression of a gene coding for its biosynthesis/degradation. Two genes coding for an L- ascorbate oxidase (VVTU23718_at, VVTU29284_at) were up-regulated at EL 35 and/or EL 36 at least in one season. Moreover, a gene coding for a L-galactono-1,4-lactone dehydrogenase (VVTU8069_at, cluster 4) which catalyzes the final step in ascorbic acid biosynthesis and a gene coding a GDP-mannose 3,5-epimerase 1 (VVTU27380_s_at) which constitutes an alternative pathway of ascorbate biosynthesis were both down-regulated at EL 36 and at EL 38 as evaluated by qPCR (Table 2, Figure 9). L-ascorbate is also a biosynthetic precursor in the formation of L-tartaric acid which also decreases during ripening. The transcript abundance of a gene involved in its biosynthesis and coding for *Vitis vinifera *L-idonate dehydrogenase (VVTU4643_at) was down-regulated, however, only in 2008 season (Table 2, Additional file 2). Recently, strong developmental regulation of ascorbate biosynthetic, recycling and catabolic genes was demonstrated in grape berries, with the ascorbate precursor being accumulate at low levels and its flux diverted towards the synthesis of tartaric acid [48].

A gene coding for a Latex cyanogenic beta glucosidase (VVTU38305_s_at) was up-regulated at EL 35 and EL 36. Grimplet and co-workers [49] found that a gene encoding cyanogenic beta glucosidase was over-expressed in the skin. Cyanogenic glycosides are glycosides of α-hydroxinitriles and their involvement in fruit ripening has been previously mentioned for strawberry [50]. The possibility that cyanogenic compounds are present in berries remains to be excluded [51]. Furthermore, a gene coding for Beta-cyanoalanine synthase (VVTU40443_s_at, cluster 8) putatively involved in cyanide detoxification was up-regulated at EL34, EL 35 and EL 36. Interestingly, a gene coding for a myrosinase precursor (VVTU6270_at) was up-regulated at EL36. Myrosinases or beta-thioglucoside glucohydrolases hydrolyze glucosinolates liberating defense compounds such as isothiocyanates and nitriles. Glucosinolate derivatives contribute greatly to the distinctive flavor and aroma of cruciferous vegetables [52].

We observed more genes up-regulated and implicated in biotic stress response during ripening in 2008 season (Additional file 6). Though environmental aspects may be involved, it can also be considered that this observation is related to the fact that the amount of skin per berry was higher in 2008, and this tissue is expected to express more genes related to defense. Such is the case of a gene coding for Anthraniloyal-CoA: methanol anthraniloyal transferase (VVTU687_at, cluster 8) that displayed remarkable increase in transcript abundance (240.6 and 373.3 fold change at EL 36 in 2007 and 2008 season, respectively). Up to our knowledge this gene has not been previously related to grape ripening and may be involved in phytoalexin synthesis in response to stress [53].

#### Flavonoid metabolism

Genes coding for enzymes acting on flavonols, stilbenes, and anthocyanins synthesis were noticed to be induced during grape ripening as previously described [16].

A gene coding for a flavonol synthase (VVTU9714_at, cluster 8) was up-regulated at EL 34, EL 35 and EL 36 displaying higher transcript abundance at this later stage. This enzyme is responsible for the conversion of dihydroflavonols to flavonols which are important co-pigments that stabilize anthocyanins in wine. On the other hand, a gene coding for a dihydroflavonol-4-reductase (VVTU20756_at, cluster 5) was down-regulated at *véraison *and ripe stages. This enzyme is responsible for the conversion of dihydroflavonols to leucoanthocyanidins which are precursors of anthocyanidins and tannins. This constitutes a difference comparing to the recently published results in Cabernet Sauvignon and Norton varieties [54]. Transcripts of dihydroflavonol-4-reductase increased to the highest levels at *véraison *in both varieties, and then declined sharply in Cabernet Sauvignon, but remained at the same levels throughout the ripening stages in Norton. As described by Pilati et al. [9] a gene coding for an anthocyanidin reductase (VVTU13083_at, cluster 5) which catalyzes the formation of epicatechin-derived compounds was also down-regulated at EL35 and EL36 since proanthocyanidins/tannins synthesis decreases after *véraison*.

Interestingly, a gene coding for Flavanone 3-hydroxylase (VVTU39787_s_at, cluster 2) was down-regulated at EL 35 but up-regulated at EL 36, and qPCR analysis further revealed up-regulation at EL 38 in both seasons (Figure 9). This suggests isoenzyme specific activation due to a switch from proanthocyanidins to anthocyanin synthesis.

It was noticed up-regulation at EL 34 and EL35 of a gene coding for UDP-glucose: anthocyanidin 5,3-*O*-glucosyltransferase with homology to a Flavonol 3-*O*-Glucosyltransferase-like protein (VVTU13618_x_at, cluster 7). Though both annotations can be correct the pattern of expression suggests that the gene is likely to code for the latter enzyme which is responsible for glucosylation of flavonol aglycones such as kaempferol, quercetin and myrecitin. In fact, in grape berry these compounds are present as the corresponding glucosides, galactosides, and glucuronides [55]. Recently, Ali et al. [20] found in Trincadeira grapes a decrease in content of quercetin glucoside following *véraison *probably due to the utilization of its precursors (dihydrokaempferol and/or dihydroquercetin) in the production of anthocyanins.

We also noticed up-regulation of a quercetin 3-*O*-methyltransferase 1 (VVTU9453_at, cluster 1) with homology to a Vitis vinifera putative O-methyltransferase that was up-regulated at EL36 reaching its peak of expression at EL38 in both seasons (Figure 9). This enzyme may be responsible for the conversion of anthocyanidins and may contribute for the varietal specific anthocyanin profile. For instance, cyanidin is converted to peonidin by the action of 3'-*O*-methyltransferase [56].

Anthocyanins provide the vibrant purple tones of red wines. The accumulation of anthocyanins in the skin of red grapes coincides with expression of the gene encoding the final step in anthocyanin biosynthesis, UDP-glucose: flavonoid 3-*O*-glucosyl transferase (UFGT). A gene coding UDP-glucose:flavonoid 3-*O*-glucosyltransferase (VVTU17578_s_at, cluster 8) displayed increased transcript abundance at EL 35 and EL 36.

Isoflavonoids comprise a class of defense compounds found mostly in legumes. Little information is available related to the involvement of isoflavonoids in grape ripening. Isoflavone reductase catalyzes the reduction of isoflavones to isoflavonones. Recently, this protein was shown to be present in embryogenic callus of *Vitis vinifera *and involved in stress response [57]. Proteomic studies revealed that a isoflavone reductase-like protein showed highest abundance before *véraison *[17]. Here we noticed the down- and up-regulation during ripening of genes coding for isoflavone reductase (VVTU13266_s_at, cluster 5, VVTU13951_at, cluster 1, VVTU12956_at, cluster 1). The latter may be involved in the synthesis of stress response-related compounds. In addition, a gene coding for a CYP81E1 Isoflavone 2'-hydroxylase (VVTU22627_at) was up-regulated at EL 36 in 2008 (Additional file 6).

#### Aroma development

Several free and bound volatiles have been reported in grapes and play a role in wine aroma. Cinnamyl alcohol dehydrogenase is involved in the synthesis of lignin precursors but cinnamyl alcohol derivatives are also responsible for fruit flavor and aroma [43]. Most genes coding for cinnamyl alcohol dehydrogenase (CAD) were down-regulated during ripening (Additional file 5), which may be related to the observed decrease in *cis*-coumaroyl derivatives and *trans*-caftaric acid when approaching *véraison *(Additional file 2). Nevertheless, one gene coding for a Cinnamyl-alcohol dehydrogenase (VVTU27826_x_at) was up-regulated at EL 35 and EL 36. A CAD gene was reported to be up-regulated during fruit ripening in strawberry and suggested to be involved in flavor development and lignification of vascular elements [43]. Another CAD gene (VVTU33502_at) displayed an interesting pattern since it was up-regulated at EL 34, just before *véraison *and down-regulated at EL36.

Multiple lipoxygenase isoenzymes have been described in plants [58]. We observed up- and down- regulation of several genes coding for lipoxygenases (Additional file 5). It is tempting to speculate that lipoxygenase isoforms activated pre-*véraison *are likely to be involved in jasmonic acid biosynthesis and cell growth, whereas lipoxygenase isoforms activated post-*véraison *may be involved in mobilization of lipids for gluconeogenesis, cell expansion and in the synthesis of C6 volatile compounds. Lipoxygenase-derived hydroperoxy fatty acids are metabolized through major pathways involving enzymes such as the hydroperoxide lyase [59]. A gene coding for fatty acid hydroperoxide lyase (HPL1; VVTU37595_s_at, cluster 7) was up-regulated at EL35. Costantini and co-workers [60] noticed in Malvasia grape berries, an increase in lipoxygenase activity, and the concomitant production of C6 compounds such as hexenol and hexanal. Recently, contents in (E)-2-Hexenal and Hexanal were shown to peak at EL36 in Trincadeira grapes (unpublished results). Hexenal can be converted to hexanol by alcohol dehydrogenases. Two genes coding for alcohol dehydrogenases were up-regulated either at EL 34 and/or EL 35 and EL 36 (VVTU4210_at, cluster 8, VVTU6090_s_at). Production of volatiles as a result of alcohol dehydrogenase activity was suggested to contribute to the development of taste and aroma in fruits [61]. Interestingly, the leaves of *Adh2 *transgenic grapevine overexpressors showed increased levels of monoterpenes, carotenoids, proanthocyanindin polymerisation and benzyl alcohol [62].

Terpenes, which are precursors for important aroma compounds accumulate at *véraison *[63]. Interestingly, a gene coding for a (-)-isopiperitenol dehydrogenase (VVTU2626_at) was up-regulated at EL 34, EL 35 and EL 36 peaking at *véraison*. This enzyme is involved in the synthesis of monoterpenoids (e.g. menthol) which are the main volatile components in essential oils. On the other hand, a gene coding for (+)-neomenthol dehydrogenase (VVTU21725_at, cluster 8) putatively involved in menthol biosynthesis, a volatile monoterpenoid, was up-regulated at EL35 and even more at EL36 in both seasons.

Some volatile terpenes are not derived directly from isoprenoid pyrophosphates but instead from the cleavage of carotenoids by carotenoid cleavage dioxygenases [64]. Three genes coding for a 9-*cis*-epoxycarotenoid dioxygenase 2 (isoenzyme carotenoid cleavage dioxygenase 1; VVTU17555_s_at, VVTU8254_at, cluster 8, VVTU650_at, cluster 7) were up-regulated at EL 35 and may contribute to the formation of the flavour volatiles [65].

Several genes putatively involved in aroma development displayed different patterns of expression between years which may be due to seasonal variation. This can lead to differences in wine aroma, though obviously a complex interplay of many other factors is involved.

One gene coding for a (-)-germacrene D synthase (VVTU13316_s_at) was down-regulated at EL 35 but only in 2008 (Additional file 6). A gene coding for a germacrene D synthase was, however, shown to be up-regulated at ripening initiation of Cabernet Sauvignon grapes [66], which highlights cultivar differences if the annotation corresponds to this specific enzymatic activity.

#### Growth regulators

Although grapes are a non-climacteric fruit, ethylene has been suggested to promote ripening by increasing modestly around *véraison *but its role is still unclear [6]. Abscisic acid, however, has a clear promoting role in grape ripening. During the earlier phases of berry development auxin and cytokinins may act to delay ripening [6]. Amongst the genes related to hormone metabolism in the core set of 7130 genes, those related to auxin and ethylene were the most represented.

##### Auxins

Though exogenous auxins can suppress or delay grape ripening [67] the role of endogenous auxin is not fully understood. In grape, it has been generally accepted that indole-3-acetic acid (IAA) levels peak after anthesis and then decline to very low levels in the ripe fruit, though other studies report relatively constant levels during grape ripening [6]. Regarding auxin biosynthesis, we found a gene coding for an indole-3-acetic acid-amido synthetase GH3.8 (VVTU3560_at, cluster 1) that was up-regulated at EL36 whereas a gene coding for a indole-3-acetic acid-amido synthetase GH3.2 (VVTU1335_at) showed a decline in expression at EL35 and EL 36. The enzyme GH3 is responsible for the formation of IAA conjugates with amino acids that may reversibly remove IAA from the active pool. In Arabidopsis, endogenous auxin content is coordinately regulated through negative feedback by a group of auxin-inducible *GH3 *genes that are involved in biotic and abiotic stress responses [68]. Recently, the GH3 catalyzed formation of IAA conjugates during ripening was suggested to represent a common IAA inactivation mechanism in climacteric and non-climacteric fruit which enables ripening to occur [67].

A transcript encoding IAA-amino acid hydrolase 1 (ILR1) (VVTU35572_s_at), which is putatively involved in IAA homeostasis, was up-regulated at EL 34, EL35 and EL36.

*Aux/IAAs *have been identified as rapidly induced auxin response genes [69]. Many genes coding for Aux-IAA proteins were down-regulated during ripening (VVTU17953_s_at, cluster 5, VVTU1813_at, cluster 6, VVTU7286_at, cluster 2, VVTU23500_at, cluster 5, VVTU2445_s_at, cluster 5) which may suggest that auxin levels are indeed lowered after *véraison*. Nevertheless, two genes coding for IAA19 (VVTU3361_at, cluster 8) and IAA16 (VVTU33878_s_at, cluster 8) were up- regulated at EL34, EL35 and EL 36.

Auxin-response factors bind auxin-response elements of auxin responsive genes and thus, seem to act as regulators of gene transcription [69]. Several auxin response factors (ARFs 1, 2, 3, 4, 6, 10, 18) were down-regulated at EL35 and EL36 or already at EL34 (Additional file 6).

Genes coding for transport inhibitor response 1 protein were up-regulated (VVTU2614_s_at) and down-regulated (VVTU7869_at) during ripening. The *TIR1 *(transport inhibitor response 1) gene encodes an F-box protein integrating the SCF complex that mediates Aux/IAA degradation [70].

A gene coding for a auxin responsive Small Auxin Up RNA protein (SAUR) 29 protein (VVTU18738_s_at, cluster 8) was up-regulated during ripening in opposition to a gene coding for Auxin-responsive SAUR31 (VVTU38338_x_at). The same was described for Cabernet Sauvignon [15]. Interestingly, a gene coding for an Auxin-responsive SAUR9 (VVTU19090_s_at) was up-regulated at EL 35 during 2007 but down-regulated during 2008. Genes coding for other auxin- responsive proteins also displayed different patterns of expression between seasons (Additional file 6).

The majority of transcripts related to auxin transport and perception displayed decreased abundance at the onset of *véraison*. Genes coding for auxin efflux carriers including PIN1 and influx carriers (VVTU16083_at, VVTU35909_s_at, cluster 5, VVTU33865_s_at, cluster 2, VVTU16124_at, cluster 6) were down-regulated at EL 34, 35 and/or EL36. The putative inhibition of polar auxin transport in ripe grapes is not so surprising since flavonoids which accumulate at high levels during ripening have been described to inhibit polar auxin transport involving PIN1 [71].

##### Ethylene

The role of ethylene in grape ripening is still not fully understood though it is generally considered to have a role in promoting ripening [6]. In fact, the application of 1-methylcyclopropene, a irreversible inhibitor of ethylene receptors, prior to *véraison *reduced berry size and anthocyanin accumulation [8]. Moreover, ethylene application at *véraison *led to an increase in berry diameter and modulated the expression pattern of ripening-related genes [72]. A small and transient increase of endogenous ethylene production was shown to occur just before *véraison *together with an increase in 1-aminocyclopropane-1-carboxylic acid (ACC) oxidase activity, the enzyme responsible for the last step in ethylene biosynthesis [8]. The protein concentration of ACC synthase was shown to peak at *véraison *in Nebbiolo Lampia berries [17].

We observed decreased transcript abundance in genes coding for ACC synthase (VVTU6382_at, cluster 6; VVTU5165_at) at EL 35 and EL 36 though one gene was up-regulated at EL34 at least in 2007 (VVTU12042_at, Additional file 6, Table 2). Several genes coding for ACC oxidase were also down-regulated during ripening (Additional file 6), and one was up-regulated (VVTU5909_at, cluster 7).

In Pinot Noir [16] the putative peak in ACC oxidase transcript accumulation occurred immediately before *véraison *and in Cabernet Sauvignon grapes at E-L stage 32 [15]. These authors however, did not identify so many genes coding for ACC oxidase as we have in this work. Our results suggest that the peak occurs before *véraison *but some isoforms of ACC oxidase may be active following *véraison*. In watermelon, a non-climacteric fruit, a homolog of ACC oxidase was also induced in ripening stages [73].

The ability to perceive, transduce and act upon hormone signals is likely to vary through development [6]. The transcript levels of some grape ethylene receptors changed during berry development [15]. Ethylene is perceived by a family of membrane associated receptors, including ETR1/ETR2 and EIN4 in Arabidopsis (reviewed by [74]). Genes coding for these receptors were up-regulated during ripening (VVTU1588_at, VVTU19389_s_at, cluster 1). A gene coding for EIN4 was recently shown to increase its expression during ripening of Muscat Hamburg grapes [9]. Using qPCR analysis we found that the gene coding for ETR1 displayed increased transcript abundance from EL35 until EL38 in both seasons (Figure 9). Ethylene levels may indeed lower during ripening since ethylene binding has been proposed to inhibit receptor function [74].

We found down-regulation at EL 35 of genes coding for EIN3-binding F-box protein 2 (VVTU2683_s_at), and at EL 35 and EL 36 for ethylene-insensitive 3 (EIN3) protein (VVTU8555_at) that shows homology to an EIL1 related protein. In Arabidopsis, there are six members of the EIN3 family, in which EIN3 and EIL1 are the most closely related proteins [74]. EIN3 is a positive regulator of ethylene responses. The nuclear protein EIN3 is a transcription factor that regulates the expression of its immediate target genes such as ERF1 [74]. This gene (VVTU8172_at, cluster 1) displayed high transcript abundance at EL 36 especially in 2008 season.

Interestingly, a gene coding for a MAP3K protein kinase (VVTU12870_s_at, cluster 1) was up-regulated at EL 36 in both seasons. The Arabidopsis MAPKs MPK3 and MPK6 seem to play a central role in the regulation of the ethylene response pathway by promoting the stabilization of EIN3 but recent investigations suggest their involvement in modulating ethylene biosynthesis rather than the signaling pathway [75].

ERF1 belongs to a large family of APETALA2-domain-containing transcription factors that bind to promoters of many ethylene inducible genes. Furthermore, ERF1 is also involved in JA mediated gene regulation [76]. A transcriptional cascade that is mediated by EIN3/EIN3-like (EIL) and ERF proteins leads to the regulation of ethylene controlled gene expression [74]. Interestingly, glucose enhances EIN3 degradation, highlighting the previously mentioned crosstalk between sugar and hormonal metabolism. Besides ERF1 other genes coding for transcription factors were up-regulated at EL35 and EL36 such as coding for ERF3 (VVTU18607_s_at, cluster 8) and for DREB sub A-5 of AP2/ERF transcription factor (VVTU17388_at). This AP2/ERF family of transcriptions factors was recently shown to be involved in grape ripening [77].

Many other genes coding for transcription factors were also down-regulated (Additional file 6) such as AP2/EREBP transcription factor (VVTU4551_at, cluster 5). Noticeably, a gene coding for an Ethylene-responsive transcription factor ERF105 (VVTU35437_at) was down-regulated during ripening in 2007 but up-regulated in 2008. Pilati and co-workers [16] also observed induction and repression of several genes coding EREBPs.

Altogether our results suggest that ethylene signaling pathways may play an important role prior to *véraison *as it has been described for other non-climacteric fruits. In watermelon, ethylene production was highest in the green fruit stage [73], and decreases in later developmental stages, similar to citrus [78] and strawberry [79]. Recently, it was suggested that a downstream portion of the ethylene-mediated signaling pathway may be activated during pepper ripening without climacteric ethylene production but via the alteration of ethylene sensitivity [80]. This may be the case in grape. It should be taken into account that a specific signaling pathway, possibly involving ERF1, is activated during grape ripening.

##### Jasmonic acid

The role of jasmonic acid in grape ripening is also poorly understood. A gene which based on genomic annotation codes for an IMP dehydrogenase (VVTU16654_a, cluster 3) was up-regulated at EL 35 and EL 36 peaking at *véraison*. Interestingly, this gene showed high homology to LEJ2 (LOSS OF THE TIMING OF ET AND JA BIOSYNTHESIS 2). The peak of expression at *véraison *in both seasons was confirmed and clearly observed by qPCR (Figure 9). Up to our knowledge this gene has not been previously reported in the context of fruit ripening. The study of this gene deserves further attention since as ethylene; jasmonic acid seems to be synthesized in lower amounts following *véraison*. In fact, several genes induced by jasmonates were down-regulated at *véraison *or at ripe stage such as EDS5 (ENHANCED DISEASE SUSCEPTIBILITY 5) (VVTU35149_at, cluster 2), phytoalexin-deficient 4 protein (PAD4) (VVTU14779_at) and cellulose synthase CESA3 (VVTU26669_at). In addition, mRNAs involved in the biosynthesis of jasmonic acid, namely those coding for allene oxide cyclase (homolog related to mangrin, VVTU7003_at), 12-oxophytodienoate reductase 3 (VVTU4246_at, cluster 6) and 12-oxophytodienoate reductase 2 (VVTU17030_s_at) were less abundant at EL 35 and EL 36. The decrease in expression of this latter gene during ripening was also reported for Cabernet Sauvignon [15]. Nevertheless, a gene coding for an allene oxide synthase (VVTU16057_at, cluster 8) putatively involved in jasmonic acid biosynthesis was strongly up-regulated at EL 35 and EL 36. One gene coding for a MYC transcription factor involved in jasmonic acid- dependent transcriptional activation was up-regulated at EL 34 just before *véraison *(VVTU34392_at, Additional file 5) whereas a gene coding for a Coronatine-insensitive 1 (COI1) related protein (VVTU23697_at, cluster 8) was up-regulated at EL 35 and EL36. COI1 is an F-box component of SCF (SKIP-CULLIN-F-box) complexes that in response to the hormone, targets JAZ (jasmonate ZIM-domain) repressor proteins for degradation [81]. Genes coding for JAZ1 and JAZ8 were up-regulated during ripening (VVTU38616_s_at, cluster 8; VVTU39811_s_at, cluster 1) whereas for JAZ3 (VVTU4273_s_at, cluster 6) was down-regulated. Interestingly, a gene coding for a JAR1-like protein (VVTU3032_at) was up-regulated at EL 36 but only in 2008 season. JAR1 encodes a jasmonic acid amino acid synthetase involved in conjugating jasmonic acid to Ile [82] which is necessary for its activation. Further studies are required to evaluate how this difference may affect grape composition in differentes seasons. Jasmonic acid and methyljasmonate are known to promote the synthesis and accumulation of resveratrol in grapevine cell cultures [83]. However, there are no reports linking endogenous jasmonates and activation of phenylpropanoid synthesis in grapes. In fact, in Trincadeira berries genes coding for jasmonate O-methyltransferase (VVTU35706_at; VVTU11913_at, cluster 6) putatively involved in the volatile methyljasmonate synthesis were down-regulated at EL 35 and EL36, suggesting that also this compound is present in lower amounts in ripe berries. On the other hand, a gene coding for a methyl jasmonate esterase (VVTU1657_s_at) putatively involved in inactivation of methyl jasmonate signaling was down-regulated.

Altogether the results suggest that though jasmonates' concentration may decrease in grapes following *véraison *they are likely to play a role in ripening possibly through interaction with other growth regulators. For instance, NPR1 is involved in the antagonistic interaction between salicylic acid and jasmonic acid [84] and the correspondent gene is up-regulated at EL36 (VVTU7560_at, cluster 1).

##### Polyamines

Polyamines are known to be involved in plant growth and differentiation and in stress/defense responses [85]. During fruit development, rates of polyamine and ethylene biosynthesis are normally opposed possibly due to the inhibitory effects of polyamines on ethylene biosynthesis and vice versa [86]. Since ethylene levels are likely to decrease following *véraison*, polyamines' levels may increase. This is suggested by the increase in transcript abundance at EL 35 and/or EL 36 of genes coding for an Arginine decarboxylase (Fragment) (VVTU12839_at, cluster 8), S-adenosylmethionine decarboxylase (VVTU12964_s_at, cluster 8), spermidine synthase (VVTU1269_s_at) and spermine synthase (VVTU5224_at, cluster 1, VVTU10365_at). These enzymes are involved in polyamine biosynthesis. Furthermore, we found that the gene coding for arginine decarboxylase kept increasing its transcript abundance up to EL 38 in both seasons (Figure 9).

Polyamines have been reported to be inducers of flowering, promoters of fruitlet abscission and involved in fruit set in grapevine [87]. However, up to our knowledge polyamines have not been suggested to play a role in grape ripening. In fact, previous studies in Cabernet Sauvignon and Pinot Noir grapes did not show up-regulation of genes coding for enzymes involved in polyamine biosynthesis [15,16]. Another enzyme involved in polyamine biosynthesis is ornithine decarboxylase but no differential expression of the correspondent gene was observed during ripening (data not shown). The intracellular free polyamine pool is affected by its synthesis and degradation among other mechanisms. Amine oxidases catabolize putrescine (diamine) and polyamines and can yield *γ*-aminobutyric acid (GABA) [88], a compound that increased in Trincadeira mature grapes (Table 1, Additional file 3). In this grape variety, we found up-regulation at EL 35 and/or EL 36 of four genes coding for amine oxidases (VVTU37047_at, cluster 1, VVTU6472_at, VVTU851_at, cluster 8, VVTU5226_at) which may indicate that an active catabolism of polyamines is occurring during ripening. Studies are undergoing to understand the role of polyamines in grape ripening.

##### ABA metabolism

Several studies report an increase in free ABA levels around *véraison *concomitant with sugar accumulation and color development [6]. Furthermore, ABA application has also been shown to induce expression of a MYB transcription factor known to coordinately activate the anthocyanin biosynthetic pathway [89]. The possibility that ABA can induce sugar uptake and accumulation as well as increase the synthesis of phenylpropanoids has led to the proposed role of ABA in promoting grape ripening [6].

Recently, the interplaying between ABA and sugar signaling pathways was shown [10] as well as between ABA and ethylene which may be required for the onset of grape ripening [9].

Two genes coding for a 9-*cis*-epoxycarotenoid dioxygenases (VVTU17555_s_at, VVTU8254_at, cluster 8) were up-regulated during ripening in both seasons though the first peaked at EL 35. This enzyme catalyzes a crucial step in ABA biosynthesis suggesting that ABA levels increase following *véraison *[90].

Besides being involved in triggering ripening, the production of ABA in grapes is likely to be related to seed development [49].

A gene coding for an ABA-responsive element-binding protein 2 (AREB2) with homology to gene grip55 was up-regulated at EL 35 (VVTU783_at, cluster 7). This protein is a transcription factor involved in control of ABA-responsive genes and it was suggested to play a role in controlling ABA-/water-stress-inducible gene expression during ripening in grape berries [91].

Interestingly, the transcript abundance of a gene UBP1 interacting protein 2a (UBA2a) with homology for a RNA-binding protein AKIP1-like protein (VVTU19049_s_at) was increased at EL 36. This protein is nuclear and involved in mRNA splicing. In *Vicia faba*, an ABA-activated protein kinase (AAPK)-interacting protein 1 (AKIP1) is phosphorylated by AAPK in response to ABA treatment. Such activated AKIP1 protein was suggested to bind other ABA-responsive transcripts such as dehydrins [92].

Many genes putatively involved in ABA signaling are up-regulated during ripening of Trincadeira grapes and have not been previously described in this context. A gene coding for OST1 (OPEN STOMATA 1) AAPK was up-regulated at EL 36 but only in 2008 (VVTU23465_at, Additional file 6).

The ABA-activated kinases were identified as SNF1-related protein kinase (SnRK) 2.2,

SnRK2.3, and SnRK2.6 (also known as OST1, the Arabidopsis ortholog of AAPK). OST1/SnRK2.6 is one of the Arabidopsis SnRK2 activated by osmotic stress besides ABA and a major, positive regulator of ABA signaling [93]. Recently, protein kinases SnRK2.2, SnRK2.3, and SnRK2.6 were suggested to have partially redundant functions but together, are essential for ABA responses whereas SnRK2-7 and SnRK2-8 play a minor role in ABA signaling [94]. A gene coding for a SnRK2-8 (VVTU12347_s_at) was up-regulated at EL 35 also only in 2008.

The seasonal differences in ABA signaling were further supported by the down-regulation of a gene coding for SNF1 PROTEIN KINASE 2-3 AKIP OST1 (VVTU22232_at) but only in 2007 (Additional file 6).

A gene coding for an ABI1 (ABA INSENSITIVE 1; VVTU28731_s_at), a PP2C-type protein phosphatase that interacts with OST1 and negatively regulates many aspects of ABA signaling [93] was up-regulated at EL 34, 35 and EL 36.

##### Brassinosteroids

Brassinosteroids (BR) have been implicated in playing an important role in berry development [7].

Pilati et al. [16] reported that the transcript abundance of a gene coding for VvBR6OX1, which converts 6-deoxocastasterone to castasterone, the only bioactive brassinosteroid detected in grape, peaked just before *véraison *in agreement with previous data [7]. In Trincadeira this gene (VVTU647_at) was down-regulated at EL 35 and EL36 and no differential expression was observed at EL 34 (at least in 2007).

In other species, a negative correlation between *VvBR6OX1 *transcript levels and the amount of the corresponding enzyme substrate was noticed [6]. This fact, could suggest that castasterone was possibly accumulating in Trincadeira berries at an earlier stage or not accumulating at any developmental stage. A gene coding for a steroid 5 alpha reductase DET2 (VVTU6606_at, cluster 6) putatively involved in brassinosteroid biosynthesis was also less expressed at EL 34, EL 35 and EL 36, suggesting that brassinosteroids' biosynthesis decrease following *véraison*.

A gene coding for an enzyme putatively involved in castasterone catabolism (CYP734A7 castasterone 26-hydroxylase) was also down-regulated at EL 35 and EL 36 (VVTU24849_at). A CYP734A7 castasterone 26-hydroxylase from tomato was shown to metabolize castasterone to 26-hydroxycastasterone and to inactivate other brassinosteroids through hydroxylation [95].

A putative brassinosteroid receptor BRI1 (BRASSINOSTEROID INSENSITIVE 1) has been described by Wang et al. [96]. Interestingly, a gene coding for BRI1 was down-regulated at EL 36 in 2007 but up-regulated in 2008 suggesting differences in perception of brassinosteroids due to different climatic conditions or eventually due to tissue specific expression. A gene coding for a transcription factor BIM1 (BES1-interacting Myc-like protein 1; VVTU14956_at) was, however, up-regulated during ripening in both seasons. The same holds true for a gene coding for a BSU1-like protein 3 BSL3 (VVTU1264_at, cluster 1) involved in brassinosteroid-mediated signalling pathway. Furthermore, we noticed a decrease during ripening in both seasons of transcript abundance of BRASSINOSTEROID-RESPONSIVE RING-H2 (BRH1) (VVTU4905_s_at). This gene is known to be down-regulated by exogenous application of brassinosteroids and in Cabernet Sauvignon grapes this transcript decreases in abundance during E-L stages 31 to 35 but increases at EL 36 [15]. This may eventually correspond to cultivar specificity.

##### Cytokinins

Cytokinins are thought to be involved in berry set and in growth promotion and tend to inhibit ripening (reviewed by [6] and references therein). The levels of zeatin are high early in grape berry development but decrease rapidly to be low at around the time of *véraison *[97]. This decrease in cytokinin levels approaching *véraison *was related to the high expression of a gene coding for a putative cytokinin oxidase before *véraison *[15]. In this work, however, we did not observe down-regulation of a gene coding for a cytokinin oxidase over berry development.

In Trincadeira grapes, the transcript levels of genes coding for cytokinin dehydrogenase 5 precursor (VVTU7035_at) and cytokinin dehydrogenase 7 (VVTU9094_s_at) putatively involved in cytokinin degradation strongly reduced at EL 35 and EL 36. A gene coding for a CR9 protein (VVTU28950_s_at), a cytokinin-repressed gene, was down- regulated following *véraison *as reported by Pilati and co-workers [16].

Several genes coding for cytokinin-O-glucosyltransferase 2 are up- or down-regulated during ripening (Additional file 5) so our data is not supportive enough of a decrease of cytokinin levels at this period.

In *Arabidopsis*, type-B response regulators (ARRs) are DNA-binding transcriptional activators that are required for cytokinin responses whereas, the type-A ARRs act as repressors of cytokinin-activated transcription [98]. Interestingly, we found a gene coding for a pseudo-response regulator 9 (APRR9) (VVTU31519_s_at) up-regulated at EL 34 in 2007 and at EL 35 in 2008. Other genes coding for type A and type B ARRs are differentially regulated at EL 34, EL 35 and El 36 (VVTU13271_s_at, VVTU9297_at, cluster 5, VVTU20270_s_at, cluster 1, VVTU9337_at).

##### Gibberellins

Evidence has been gathered that supports a role for gibberellins during fruit set (including an important role in seed development) but there is no strong evidence that gibberellins are directly involved in the control of berry ripening, though they are thought to contribute to cell enlargement [6].

Two genes coding for Gibberellin oxidase were up-regulated at EL 35 and EL 36 (VVTU13918_at, cluster 8; VVTU12369_at, cluster 8) but others are down-regulated (VVTU8591_at, VVTU9124_at, cluster 5, VVTU7332_at) at the same stages, making it difficult to understand how gibberellins catabolism occurs during ripening. In addition, several genes coding for Gibberellin-responsive and Gibberellin regulated proteins were up or down-regulated during ripening (Additional file 6).

On the other hand, a gene encoding Gibberellic acid receptor GIDL2 (VVTU1752_at, cluster 8) displayed increased transcript abundance at EL 35 and EL 36, especially in 2007. The transcript abundance of two putative Gibberellic acid receptors, GIDL1 and GIDL2, was shown to increase during development of Cabernet Sauvignon grapes [15]. In Trincadeira grapes, at EL 36, we also found up-regulation of a gene coding for Gibberellin receptor GID1L1 (TU15195_at, cluster 1) but with higher transcript abundance in 2007 season. This may be due to the fact that there was a higher cell enlargement in the berries grown in 2007.

##### Signal transduction

In this study, besides the transcription factors already reported we have identified other members of the MYB, MADS-box, NAC, basic helix loop helix (bHLH) and WRKY families and homeotic and development specific genes among others as referred for Pinot Noir berries [16]. Many transcription factors were significantly modulated in only one season what might be due to the different environmental factors or affected by the different tissue composition of the berries when they have differential patterns of expression.

It has been referred that regulation of flavonoid synthesis occurs mostly via coordinated transcriptional control of structural genes by the interaction of DNA-binding R2R3 MYB transcription factors, WD40 proteins, and MYC-like basic helix loop helix (bHLH) [99]. Recently, the Grapevine R2R3-MYB Transcription Factor 1 VvMYBF1 was shown to regulate flavonol synthesis in developing grape berries [100].

We found up-regulation of genes coding for VvMYBA1 and VvMYBA3 (VVTU17547_at, VVTU17564_s_at, cluster 8) at EL 36. In grapes, some MYB genes have been shown to be involved in flavonoid metabolism. In particular, many white grape cultivars arose from multiallelic mutations of the MYBA1 and MYBA2 genes [101], which regulate the reaction catalyzed by UDP-glucose flavonoid 3-O-glucosyltransferase that stabilizes anthocyanidins through glycosilation. MYBA2 was not represented in the chip. The transcription factor VvMYBPA1 was shown to regulate proanthocyanidin synthesis [102]. Thus, not surprisingly, this gene was down-regulated at EL 35 and EL 36 (VVTU3046_s_at). Recently, the expression pattern of a gene coding for VvMYBPA1 was shown to be strikingly different in Cabernet Sauvignon and Norton grapes showing that flavonoid pathways are regulated by different MYB factors [54]. Interestingly, a gene coding for a myb TKI1 (TSL-KINASE INTERACTING PROTEIN 1; VVTU9543_at, cluster 1) not previously described for grape ripening was up-regulated at EL35 and kept increasing up to EL36. This myb domain protein interacts with the TOUSLED (TSL)-like nuclear protein kinase that was suggested to play a role in chromatin metabolism [103].

Two genes coding for MADS box transcription factors were up-regulated during ripening in both seasons (VVTU18199_s_at, cluster 8, VVTU11835_at, cluster 7) though many genes of this family were down-regulated together with LIM-like proteins (Table 2). One gene coding for LIM domain protein WLIM1 was strongly down-regulated at EL 35 and even more at EL 36 (VVTU3258_at). The same decrease in a LIM transcription factor was observed during ripening of pepper, also a non-climacteric fruit [80].

We found up-regulation of a gene coding for a scarecrow-like transcription factor 8 (SCL8; VVTU27392_s_at, cluster 8) at EL 35 and EL 36 in both seasons whereas a gene coding for scarecrow-like transcription factor 9 (SCL9; VVTU37071_at) was up-regulated at EL 36 only in 2008 season (Additional file 6). Scarecrow-like proteins have been suggested to be involved in ripening of pineapple together with zinc finger proteins [44]. One gene coding for a zinc finger (C3HC4-type RING finger; VVTU3183_at) was up-regulated only at EL35 in both seasons. This transcription factor may play an important role as a turning point into the maturation stage.

Transcription factor analysis revealed the induction of many WRKY genes at *véraison *and some showed a ripening-specific profile (Additional file 5). These transcription factors have been shown to participate in the regulation of plant defense responses, developmental programs and fruit maturation [104]. Two genes coding for a WRKY DNA-binding protein 48 and 23 (VVTU40803_s_at, VVTU2080_at, cluster 8) were up-regulated during ripening in both seasons starting increasing their transcript abundance already at EL 34 (at least in 2007).

The majority of transcripts with homology to NAC transcription factors appeared modulated in a positive way in the study interval (Table 2, Additional file 5). These transcription factors family are involved in biotic and abiotic stress responses, fruit development, ABA signaling and many other processes [105]. In ripening of watermelon fruits NAC protein homologs were suggested to play a role in vascular differentiation [73]. In these fruits bZIP transcription factors were also showed to be involved in ripening as it is indicated by the results we have obtained in Trincadeira grapes. Some of the genes coding for bZIP transcription factors were up-regulated only at EL34 and EL 35 (VVTU11917_at) and others showed a ripening specific profile (VVTU5563_at, cluster 8, VVTU27362_at, cluster 8, Table 2). This class of transcription factors has been, together with those involved in MADS box regulation, implicated in both climacteric (tomato, peach) and non-climacteric (watermelon, pepper, strawberry, pineapple) fruit ripening [44,73,80,106-108].

In Arabidopsis, DOF-type transcription factors were shown to be involved in the regulation of phenylpropanoid metabolism [109]. Interestingly, a gene coding for a Dof zinc finger protein DOF3.5 (VVTU3691_at) was up-regulated only at EL35 in both seasons and may be involved in the onset of ripening.

During ripening of Cabernet sauvignon grapes a large number of genes with functions related to calcium sequestration, transport and signaling displayed developmentally regulated expression patterns [15]. A gene coding for a Calcium-dependent protein kinase (CDPK) 32 cpk32 (VVTU2538_at, cluster 7) was up-regulated at EL 35 at both seasons whereas a gene coding for another CDPK-related kinase (VVTU24659_at, cluster 2) displayed an interesting profile due to being down-regulated at EL 35 and up-regulated at EL 36. These kinases are calcium- regulated and their tissue specific expression is affected by several stimuli such as drought stress, hormone treatment, and pathogens [110].

Some CDPKs specifically interact with calcium sensor proteins CalcineurinB-like (CBLs) and for this reason are named CBL-interacting protein kinases (CIPKs). Recently, a grapevine Shaker inward K+ channel activated by the CBL1-CIPK23 network was shown to display strong up-regulation upon drought stress [111]. Eleven genes coding for CIPKs were differentially expressed during ripening (Additional file 6). Interestingly, a gene coding for a CBL-interacting protein kinase 1 (CIPK1) was up-regulated at EL 35 in both seasons (VVTU13369_at) and may eventually make part of an important signaling module associated with the onset of ripening.

With no lysine (WNK) protein kinases and Ste (sterile) 20 kinases are essential for survival after hypertonic shrinkage of C. elegans [112]. Two genes coding for STE20/SPS1 proline-alanine-rich protein kinase (VVTU26057_at, cluster 8, VVTU30962_at, cluster 8) displayed increased transcript abundance from EL 35 to EL 36, and are putatively involved in osmoregulation during grape ripening. Up to our knowledge these genes have not been related to fruit ripening.

Receptor like kinases (RLKs) have been implicated in various signaling pathways, including brassinosteroid perception and plant defense. Recently, a novel Lec-receptor kinase-like protein in lemon was identified in response to fungi infection [113]. During ripening of Trincadeira grapes genes coding for several types of RLKs were significantly modulated. This was the case of wall-associated kinases (WAKs) which are tightly bound to the cell wall and are required for cell expansion during plant development (reviewed by [114]). So it is not surprising that genes coding for a WAK receptor protein kinase (VVTU9861_at, cluster 8) and a wall-associated kinase 4 (VVTU38545_at, cluster 1) were up-regulated during ripening stages (Table 2) when cell expansion occurs in the berry.

Importantly, a gene coding for a Receptor protein kinase (VVTU11578_at, cluster 7) presented a peak of expression at EL35 in both seasons and is eventually involved in promoting ripening.

Moreover, we have identified four genes coding for receptor protein kinase PERK1 that were up-regulated at EL 36 (VVTU9535_at, cluster 8, VVTU8084_at, cluster 1, VVTU4451_at, VVTU10748_at). Two of these displayed increased transcript abundance already at EL 35 and increased further at EL 36 (VVTU9535_at, cluster 8, VVTU10748_at). RLK candidates with similarity to AtPERK have been previously identified during ripening of grapes [66] and watermelon [73].

##### Light signaling and circadian clock

Several genes involved in the circadian rhythm oscillatory system were differentially expressed at EL 35 and/or EL 36 what suggests that light plays a role in regulating the ripening process (VVTU2126_at, cluster 1, VVTU5883_at, VVTU2284_at, cluster 1, VVTU2454_s_at, Additional file 6). A gene coding for an ELIP1 (EARLY LIGHT-INDUCIBLE PROTEIN) was up-regulated at EL35 in both seasons (VVTU40867_x_at). During ripening of tomato fruit, the early light-inducible protein gene is expressed during the chloroplast-to-chromoplast transition [115]. Early light-inducible proteins are known to accumulate in chloroplasts during thylakoid biogenesis and under stressful conditions.

Several genes coding for transcription factors of the Constans-like family were either positively or negatively modulated during ripening (Additional file 6). A gene coding for an early flowering (ELF) 3 (VVTU2284_at, cluster 1) was up-regulated at EL 36 in both seasons. ELF3 nuclear protein is an evening-specific repressor that represses light input to the circadian clock. Its activity is thought to be required by the core oscillator to produce circadian rhythms regulating growth responses [116]. A gene coding for the MYB transcription factor CCA1 (CIRCADIAN CLOCK ASSOCIATED 1) was down-regulated during ripening (VVTU3515_s_at, cluster 6). This is not in agreement with what was obtained for Cabernet Sauvignon grapes where a transcript encoding CCA1 increased in abundance at EL36 [15]. This can be due to cultivar specificities or different harvesting conditions. On the other hand, a gene coding for a timing of CAB expression 1 protein (TOC1_2; VVTU22197_at, cluster 8) from the two-component signal transduction system was up-regulated at EL 36 in both seasons.

##### Epigenetic factors, RNAi and transposons

The involvement of epigenetic factors and transposons in promoting grape ripening has been little explored. However, the expression patterns of several genes involved in chemical modification of DNA and coding for histones (Table 2, Additional file 6) indicate that epigenetic factors are involved in the onset of *véraison*. Genes coding for histones H3, H2B, H1 and H2AXb HTA3 were up-regulated during ripening in both seasons (Table 2, Additional file 5). Two genes coding for histone acetyltransferase ELP3 and HAC1 (VVTU8618_at, cluster 1, VVTU5223_at) were up-regulated at EL 36, and at EL 35 and EL 36, respectively, with the latter increasing in transcript abundance during ripening (Table 2, Additional file 6). Four genes coding for histone deacetylase and SIN3 component of histone deacetylase complex were also modulated during ripening though displaying different expression patterns (VVTU5815_at, cluster 1, VVTU87_at, cluster 4, VVTU3690_at, cluster 8, VVTU16981_at), which may be related to their specific functions. Recently, the expression pattern of genes coding for histone acetyltransferases and histone deacetylases was studied in several grapevine organs, and suggested specific roles for these enzymes in regulating transcriptional activity during grape ripening [117].

Three genes coding for chromatin-remodeling proteins (VVTU32711_at, VVTU11309_at, VVTU38460_at) displayed different expression profiles and tend to be more expressed in 2008 season (Table 2, Additional file 6). In fact, tissue-specific epigenetic modifications during fruit ripening can be expected as occurs in tomato which shows tissue-specific variations of DNA methylation [118]. Moreover, environmental stresses which are season dependent induce genetic and epigenetic changes that trigger DNA methylation [119]. A global decrease in DNA methylation during grape ripening as reported for tomato [118] is suggested by the down-regulation of a gene coding for a cytosine methyltransferase (DRM2, VVTU8524_at, cluster 6) and up-regulation of a gene coding for a DNA-3-methyladenine glycosidase I (VVTU2258_at) at the onset of ripening during both seasons. This latter enzyme acts as a base excision repair enzyme by severing the glycosylic bond of damaged bases. Moreover, *de novo *cytosine methylation in *Arabidopsis thaliana *involves components of the RNAi complex such as RNA-DEPENDENT RNA POLYMERASE 2 (RDR2), DICER-LIKE3 (DCL3), and putative SNF2-containing chromatin remodeling protein DRD1 [119]. The genes coding for these proteins were down-regulated during ripening of Trincadeira grapes but only in 2007 season whereas a gene coding for an argonaute protein was down-regulated in both seasons (VVTU5485_s_at, Additional file 6). This suggests that RNA-mediated epigenetic modifications during grape ripening may be season dependent and/or tissue specific. Interestingly, two genes involved in pre-mRNA splicing, an important mechanism of regulation of gene expression, were up-regulated during ripening (VVTU11603_at, cluster 8, VVTU28953_s_at, cluster 8).

Transposable elements can play an important role in generating both genetic and epigenetic methylation changes [119]. Nine retrotransposons (transpose by an RNA intermediate) were modulated during ripening and some showed different expression profiles between seasons (Table 2, Additional file 6) which can be due to environmental cues. In fact, most plant transposable elements are activated by different biotic and abiotic stresses [120].

Genes coding for unclassified retrotransposon proteins (VVTU15783_at, cluster 1 VVTU14689_at), a retrotransposon protein of Ty1-copia subclass (VVTU10989_at), a retrotransposon protein of Ty3-gypsy subclass (VVTU13723_x_at, cluster 7) a transposon protein of the CACTA super family and En/Spm sub-class (VVTU12696_at), transposon proteins (VVTU37074_at, cluster 1; VVTU6149_s_at, cluster 3) and transposase (VVTU5491_at, cluster 1) may play an important role in ripening since they were up-regulated at EL 35 and/or EL 36 in both seasons.

## Conclusions

This work described a comprehensive analysis of the transcriptome and metabolome during ripening of Trincadeira grapes. The combined analysis of transcripts and metabolites contributed to the elucidation of many aspects of carbohydrate, amino acid and phenylpropanoid' metabolisms during ripening. Differences have been encountered in the pattern of expression of many genes in relation to what has been published for other varieties as well as differences between years of grapes' production. For instance Trincadeira is known to contain less phenylpropanoids than other Portuguese cultivars [20] what may be related to a different primary metabolism as suggested here by an increase in sucrose as well as down-regulation of a gene coding for sucrose synthase during ripening that does not seem to occur in Cabernet Sauvignon grapes. In addition, differential expression of sugar kinases might be responsible for differences in metabolism among grapevine varieties during ripening and eventually among seasons. In particular, glucose was higher during 2008 season at EL 38 comparing to 2007 whereas sucrose and malate showed an opposite trend and succinic acid showed no significant differences. Such balance between the two sugars and organic acids may depend upon climatic conditions and represent differences in the pool of precursors for synthesis of secondary metabolites.

Good correlations were found for the content of aminoacids such as methionine, proline and glutamate and genes involved in their biosynthesis/degradation. The same holds true for the tripeptide glutathione and for organic acids such as ascorbate, succinate, tartrate, as well as phenolic compounds such as quercetin glucoside and caftaric acid. It is also worth noting the expression of genes coding for a gamma-aminobutyric acid transporter and a glutathione-conjugate transporter during ripening in both seasons. To our knowledge these transporters have not been previously described in the context of grape ripening.

Compared to other cultivars, differences have been encountered in Trincadeira regarding the flavonoid and terpenoid pathways, namely on the expression of genes coding for dihydroflavonol-4-reductase and (-)-germacrene D synthase which ultimately may have impact in specific wine characteristics.

A detailed analysis of growth regulators' metabolism and signaling pathways is provided due to their importance as possible biotechnological targets for grape ripening control. Novel information (e.g. expression of genes coding for transcription factors, receptors, diverse components of signaling pathway and metabolism) was provided for all classes of growth regulators and differences were noticed comparing to other cultivars as well as between years of Trincadeira growth. These differences certainly deserve being subjected to a more detailed study including measurements of growth regulators'content and eventual future functional analysis. Moreover, we have addressed the putative role played by epigenetic factors and transposons in grape ripening, a subject that has been very little explored.

All this information benefited from the improvements on gene annotation based on 12X coverage grapevine genome sequence assembly and also on the use of GRAPEGEN GenChip that covers approximately 50% of the Vitis genome, being more representative than previous made available Affymetrix Vitis microarrays.

Finally, our findings provide the first comprehensive transcriptomic and metabolomic study of grape ripening run over two seasons and provide a valuable contribution for the understanding of the mechanisms regulating the complex process of grape ripening.

## Methods

### Sample collection and RNA extraction

Four biological replicates (each including 80-100 berries from 8-10 Trincadeira cultivar plants) were collected around 10 a.m. in 2007 and 2008 at Plansel's vines located in Montemor-o-Novo (Southern Portugal). Samples corresponding to the developmental stages of EL 32, 34, 35, 36, and 38 (E-L refers to the modified Eichhorn and Lorenz developmental scale as described by [4] were immediately frozen in liquid nitrogen and transported to the lab in dry ice. Each biological replicate contained berries from a single row of plants, and from the sunny and shady sides of the plants. Rows distant 3 to 10 m were used.

Grapes were grinded in liquid nitrogen, seeds removed, and then RNA extraction was carried out using the extraction buffer described by [121] with additional 0.8% PVP-40. Samples were then vortexed and extracted twice in chloroform/isoamylalcohol (24:1, v/v). To precipitate proteins a KCl 2 M solution was added to the supernatant up to a final concentration of 160 mM, and samples were allowed to stay on ice for one hour. Following a centrifugation, supernatant was precipitated with 1/10 vol sodium acetate 3 M and 0.8 vol of cold isopropanol in a Corex tube, followed by washes in 70% ethanol and dissolved in water. Samples were then centrifuged before precipitation overnight on ice with LiCl 4 M, followed by washes with ethanol and then samples were dried and dissolved in water. A precipitation for 1 h on ice with KAc 2 M was then carried out for polysaccharides removal. A DNAse treatment was performed according to the suppliers' instructions (Invitrogen, San Diego, CA, USA). Samples were then extracted in phenol/chloroform/isoamylalcohol (75:24:1, v/v/v), precipitated with sodium acetate and ethanol, washed in 70% ethanol and dissolved in water. RNA was further purified using RNeasy Plant Mini kit (Quiagen, Valencia, CA, USA).

### Target preparation and hybridization of oligo arrays

RNA quality was checked using the Agilent 2100 Bioanalyzer (Agilent technologies, Palo Alto, CA). cDNA was synthesized from 4 μg of total RNA using One-cycle target labeling and control reagents (Affymetrix, Santa Clara, CA) to produce biotin labeled cRNA which was then fragmented at 94°C for 35 min into 35-200 bases in length.

Three biological replicates were independently hybridized to the GrapeGen 520510F array (Affymetrix, Santa Clara, CA). Each sample was added to a hybridization solution containing 100 mM 2-(N-morpholino) ethanesulfonic acid, 1 M NaCl, and 20 mM of EDTA in the presence of 0.01% of Tween-20 to a final cRNA concentration of 0.05 μg/ml. Hybridization was performed for 16 h at 45°C. Each microarray was washed and stained with streptavidin-phycoerythrin in a Fluidics station 450 (Affymetrix) and scanned at *1.56 *μm resolution in a GeneChip^® ^Scanner 3000 7G System (Affymetrix).

### Data and sequences analysis and gene annotation

Robust Multi-array Analysis (RMA) algorithm was used for background correction, normalization and expression levels summarization [122]. Next, differential expression analysis was performed with the Bayes t-statistics from the linear models for Microarray data (limma), included in the affylmGUI package. P-values were corrected for multiple-testing using the Benjamini-Hochberg's method [26]. Data obtained from hybridization of GrapeGen chips were filtered considering an absolute fold change ≥ 1.5 and corrected p value < 0.05.

The probesets sequences were blasted against the genes predicted from the genome (blastn, e-value < e-20, minimum of 100 bp alignment) available at the NCBI website. Gene annotation was performed by updating the annotation performed in [123] following the same protocol as described by the authors to the new genes from the 12X coverage release of the genome assembly. The genes were then assigned to functional categories according to their function. Categories have been constructed by completing MIPS functional categories plant-specifics with GO terms.

### Clustering of Expression Pattern

Median values of logExperiment Fluorescence and logControl Fluorescence from three biological replicates (control corresponds to green berries-EL 32) were used for cluster analysis. This analysis was performed using the Multiple Experiment Viewer version 4.6.2 software package, and based on the *k*-means method using Pearson's correlation distance calculated on the gene expression profiles obtained for EL 32, EL 35 and EL 36 in both years.

### Metabolic profiling using ^1^H NMR, J-resolved, COSY, and multivariate analysis

Grapes were frozen and grinded in liquid nitrogen (seeds removed with a pincet) and lyophilized for at least 72 h at -40°C. Fifty mg of material was used for each sample extraction according essentially to [124]. KH_2_PO_4 _was added to D_2_O (99.00%, Cambridge Isotope Laboratories, Miami) as a buffering agent. The pH of the D_2_O for NMR measurements was adjusted to 6.0, using a 1N NaOD solution (Cortec, Paris).

Samples were solved in 750 μl of KH_2_PO_4 _with 0, 1% trimethyl silane propionic acid sodium salt (standard purchased from Merck, Darmstadt, Germany) and 750 μl of methanol-*d4 *(99.8%, Cambridge Isotope Laboratories, Miami). Then, samples were briefly vortexed, sonicated for 10-20 min and centrifuged for 10 min at 13000 rpm. The supernatant (800 μl) was then used for analysis.

^1^H NMR and 2D *J*-resolved spectra were recorded at 25°C on a 500 MHz Bruker DMX-500 spectrometer according to [124]. The resulting spectra were manually phased and baseline corrected, and calibrated to TSP at δ 0.0, all using XWIN NMR (version 3.5, Bruker). The ^1^H NMR spectra were automatically reduced to ASCII files using AMIX (version 3.7, Bruker Biospin). Spectral intensities were scaled to TSP and to total intensity and reduced to integrated regions of equal width (0.04 ppm) corresponding to the region δ = 0.40- 10.00. The region of δ = 4.70- 5.10 was excluded from the analysis because of the residual signal of water. PCA analysis was carried out with the SIMCA-P software (version 11.0; Umetrics, Umea°, Sweden). The Pareto scaling method was used, which gives each variable a variance numerically equal to its standard deviation. Excel files containing spectral intensities reduced to integrated regions of equal width (0.04 ppm) were used for Kruskal-Wallis and Wilcoxon rank sum tests in order to determine which samples have significantly different amounts of certain metabolites.

Two dimensional NMR experiments (J-resolved, COSY, and HMBC) were measured following the parameters of our previous experiments [124].

### Anthocyanins and glutathione quantification

Grapes were frozen in liquid nitrogen, seeds removed, freeze- dried for 72-96 h at - 40°C and then 20-60 mg of the powder extracted in 1, 5 ml TFA (Trifluoroacetic acid)/methanol/H2O (0.05/80/20, v/v/v). Samples were vortexed for 1 min and then anthocyanins were extracted for 1 h on ice in Eppendorf tubes. The mixture was then centrifuged for 30 min at 13000 rpm at 4°C. A 100 μL of this sample was diluted to 1 ml in extraction solution. The solution was mixed and allowed to sit for 5 min before reading the absorbance at *A520*. Total relative anthocyanin concentration was expressed as the absorbance value at 520 nm/g of freeze-dried weight.

For glutathione quantification samples collected and lyophilized as described above were extracted in 0.5 M perchloric acid in phosphate buffer saline on ice and centrifuged for 10 min at 4°C. Total glutathione was determined using the glutathione reductase enzymatic assay [125], following the rate of absorption change at 412 nm for 15 min. Briefly, the assay was performed in a 1 mL reaction volume with 0.1 M potassium phosphate buffer, 5 mM EDTA (pH 7.5), 2U of yeast glutathione reductase (Sigma), DTNB, NADPH and 20 μL of previously neutralized extract with KOH. Glutathione content was determined based on a standard curve. All the assays were performed using an Agilent HP 8453 diode array spectrophotometer, with temperature control and magnetic stirring in the cuvette.

### Quantitative RT-PCR

Complementary DNA was synthesized from 1.5 μg RNA using a RevertAid™ H Minus M-MuLV Reverse Transcriptase (Fermentas, Burlington, Canada) according to the manufacturer's instructions. Primers' sequences (Additional File 7) were selected using Primer express software3.0 (Applied Biosystems, Forster City, CA). Real-time PCR reactions were prepared using Maxima™ SYBR Green qPCR Master Mix (2X) (Fermentas, Burlington, Canada) and performed using the StepOne™ Real-Time PCR System (Applied Biosystems, Foster City, CA). Cycling conditions were 95°C for 20 min, then 40 cycles of 95°C for 1 min and 60°C for 20 min. Expression was determined for duplicate biological replicates and triplicate technical replicates using a serial dilution cDNA standard curve per gene. Data were calculated from the calibration curve and normalized using the expression curve of actin gene (VVTU17999_s_at) that presented absolutely no differential expression in the microarray analysis.

## Competing interests

The authors declare that they have no competing interests.

## Authors' contributions

AMF designed the experiment and wrote the manuscript, sampled material, performed RNA extractions, analysis and interpretation of microarray data, performed anthocyanins quantification, starch staining, and participated in metabolomics, glutathione quantification and cluster analysis. PAR designed the primers, performed qRT-PCR and participated in data presentation. MSS participated in glutathione quantification and data presentation. KA, FM, YHC participated in metabolomics. LS carried out the statistical analysis. JG performed genomic annotation. KA, YHC, JG, JMMZ, RV, and MSP critically revised the manuscript. All authors approved the final manuscript.

The microarray data were submitted to Gene Expression Omnibus (NCBI) and are accessible through GEO accession number GSE28779.

## Supplementary Material

Additional file 1 **Weather conditions from April to September in 2007 and 2008 seasons**. Click here for file

Additional file 2 **Metabolism of organic acids and phenolic compounds**. Relative quantification of tartaric acid, citric acid, acetic acid, *cis*-coumaroyl derivatives and *trans*-caftaric acid is based on characteristic chemical shift (*δ *4.50, *δ *2.93,, *δ *1.91, *δ *7.02 and *δ *6, 38, respectively), and corresponding peak intensity. Expression levels of genes coding for *Vitis vinifera *L-idonate dehydrogenase (VVTU4643_at), and cinnamyl alcohol dehydrogenases (VVTU14855_at, VVTU21888_at, VVTU11923_at) was based on results of microarrays. *Accounts for a contamination of a spectrum corresponding to EL 32 sample collected in 2008 around *δ *1.91. Click here for file

Additional file 3 **Wilcoxon Rank sum and Kruskal-Wallis statistics applied to metabolomics data**. Click here for file

Additional file 4 **COSY analysis in a sample from 2007 corresponding to EL 35 (*véraison*)**. Spectrum is shown in the range of *δ *6.0 to *δ *8.0 ppm which is enriched in phenolic compounds. Click here for file

Additional file 5 **Core set (7130 probesets) and conserved set (3451 probesets) of modulated genes during ripening**. Information concerning fold change values, expression profile cluster, annotation, functional category and their distribution within clusters is provided. Click here for file

Additional file 6 **List of entire modulated gene set**. Annotations based on the genome and based on EST-homology are provided. Separate lists of probesets differentially expressed at each year are included. All the information is made available in 6 sheets. Click here for file

Additional file 7 **List of primers used in real time reverse transcription-polymerase chain reaction**. Click here for file
