# Supplementary figures and images for: Transcript and metabolite analysis in Trincadeira cultivar reveals novel information regarding the dynamics of grape ripening
Source: BMC Plant Biol. 2011 Nov 2;11:149. doi: 10.1186/1471-2229-11-149 (PMC3215662; doi:10.1186/1471-2229-11-149)

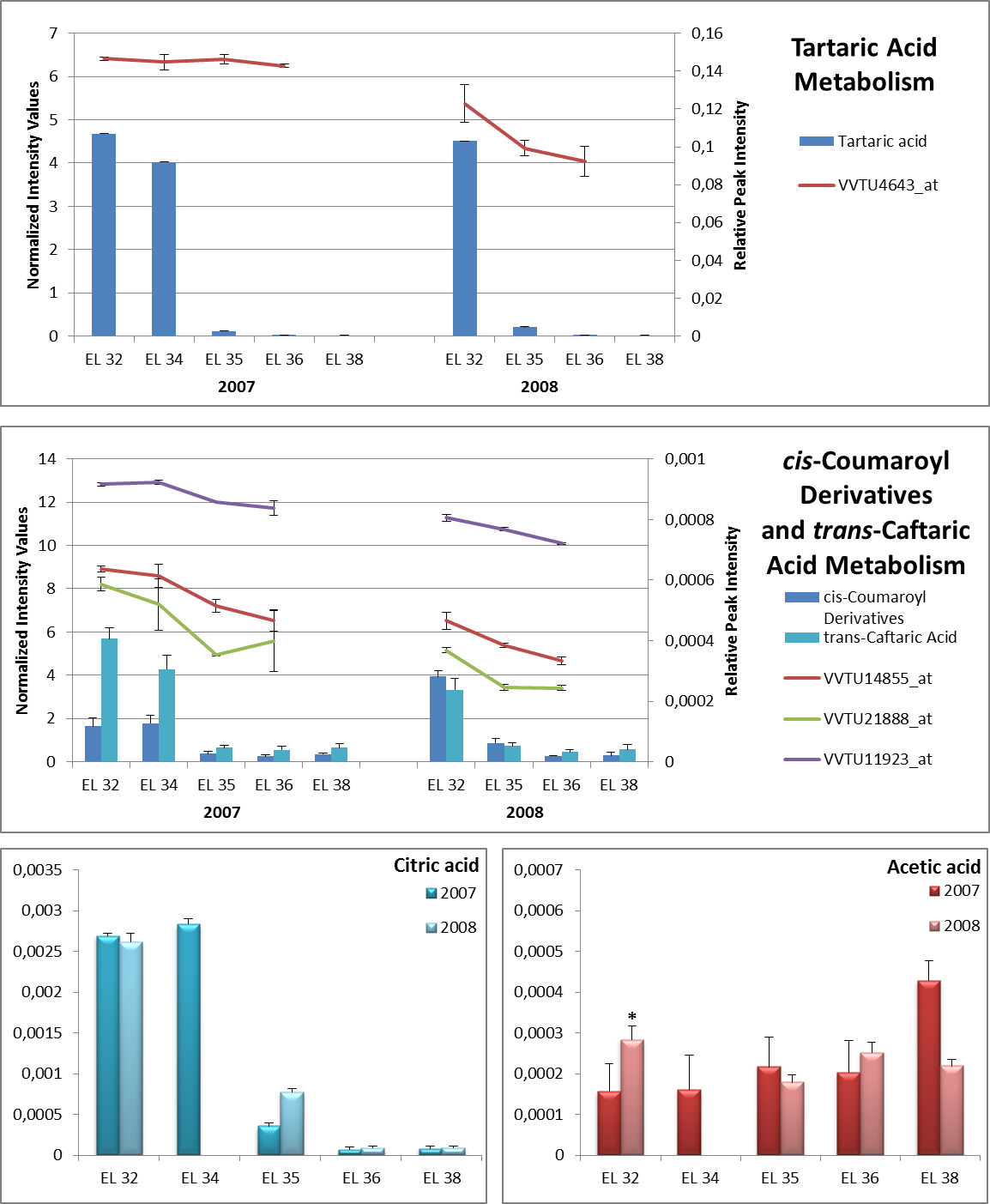

Supplement: Additional file 2 — Metabolism of organic acids and phenolic compounds. Relative quantification of tartaric acid, citric acid, acetic acid, cis-coumaroyl derivatives and trans-caftaric acid is based on characteristic chemical shift (δ 4.50, δ 2.93,, δ 1.91, δ 7.02 and δ 6, 38, respectively), and corresponding peak intensity. Expression levels of genes coding for Vitis vinifera L-idonate dehydrogenase (VVTU4643_at), and cinnamyl alcohol dehydrogenases (VVTU14855_at, VVTU21888_at, VVTU11923_at) was based on results of microarrays. *Accounts for a contamination of a spectrum corresponding to EL 32 sample collected in 2008 around δ 1.91. [file 1471-2229-11-149-S2.TIFF]

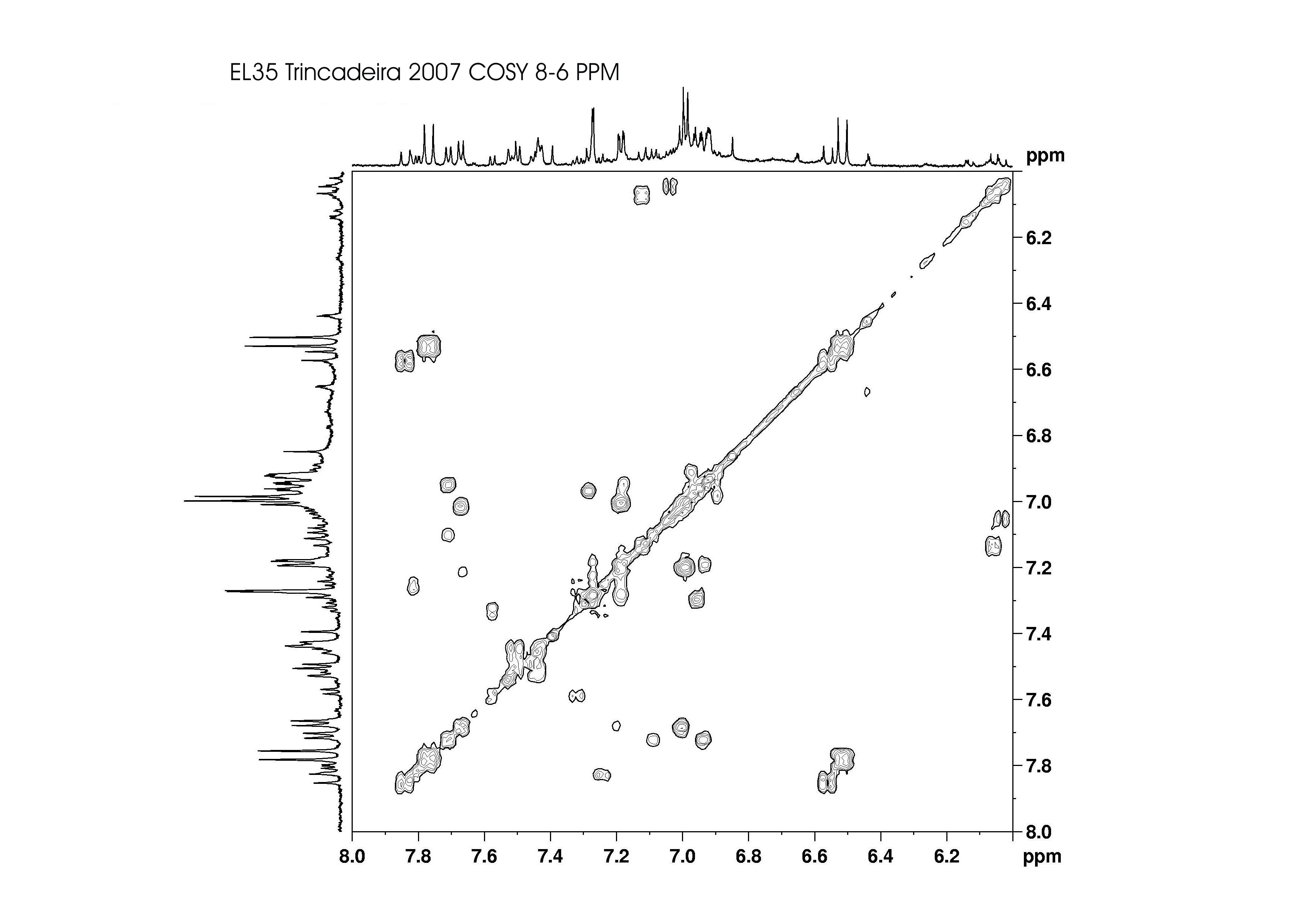

Supplement: Additional file 4 — COSY analysis in a sample from 2007 corresponding to EL 35 (véraison). Spectrum is shown in the range of δ 6.0 to δ 8.0 ppm which is enriched in phenolic compounds. [file 1471-2229-11-149-S4.JPEG]
