# Supplementary material for: Transcript and metabolite analysis in Trincadeira cultivar reveals novel information regarding the dynamics of grape ripening
Source: BMC Plant Biol. 2011 Nov 2;11:149. doi: 10.1186/1471-2229-11-149 (PMC3215662; doi:10.1186/1471-2229-11-149)
Supplement: Additional file 7 — List of primers used in real time reverse transcription-polymerase chain reaction. [file 1471-2229-11-149-S7.DOCX]

| **Annotation-Identity based on genomic annotation** | **Probe ID** |  | **5’ to 3’** | **Tm** | **% GC** | **Length** |
| --- | --- | --- | --- | --- | --- | --- |
| **Actin** | VVTU17999_s_at | Fwd | **GGTCAACCATGTTCCCTGGTATT** | 59.8 | 48 | 23 |
|  |  | Rev | **GGAGCAAGAGCAGTGATTTCCTT** | 59.9 | 48 | 23 |
| **Arginina decarboxylase (ADC)** | VVTU12839_at | Fwd | **CATCGTGGAAGATGTCGTGAA** | 58.2 | 48 | 21 |
|  |  | Rev | **ACCAGCTTCGAGGCCAAAC** | 58.9 | 58 | 19 |
| **L-galactono-1,4-lactone dehydrogenase (LGDH)** | VVTU8069_at | Fwd | **TGGAGAAAGTCAGAGGGATACAGA** | 58 | 46 | 24 |
|  |  | Rev | **CACCACAATCAAATCCCAAGATT** | 58.7 | 39 | 23 |
| **Gamma-glutamylcysteine synthetase (GCS)** | VVTU4990_at | Fwd  Rev | **TGGAATGTCCTTCCGGGA ATTCACCAGGAAGTGAAGGAAGTT** | 58.4  58.3 | 56  42 | 18  24 |
| **Ethylene receptor 1 (ETR1)** | VVTU1588_at | Fwd | **TCTCGCGCGCCAGATT** | 58.7 | 63 | 16 |
|  |  | Rev | **ACCTGTACACGCAAATAAAAGTGATT** | 58.3 | 35 | 26 |
| **IMP dehydrogenase (IMDDH)** | VVTU16654_at | Fwd | **AAGTACCGTCGGTTGCCTGTA** | 58.1 | 52 | 21 |
|  |  | Rev | **TTCCCCTTGTGACAATTCCAA** | 58.9 | 43 | 21 |
| **Succinic semialdehyde dehydrogenase (SSADH1)** | VVTU35625_s_at | Fwd | **TAGAGGCTTGTGCTTGGATTTTT** | 58.1 | 39 | 23 |
|  |  | Rev | **CGCAACCCCGCAACA** | 58.1 | 67 | 15 |
| **Quercetin 3-O-methyltrans-ferase 1 (OMT1)** | VVTU9453_at | Fwd | **ATGTAGGCTTGATCGGTATTGAACT** | 58.2 | 40 | 25 |
|  |  | Rev | **AGAAACACAATCTGCTGAGATGATAAA** | 58.5 | 33 | 27 |
| **Flavonone- 3-hydroxylase (F3H)** | VVTU39787_at | Fwd | **ACCAGAAGGTTGTGGTCAATTTCT** | 59 | 42 | 24 |
|  |  | Rev | **GATGGTTCCCGGATCGGTAT** | 59,4 | 55 | 20 |
